# From ORYZA2000 to ORYZA (v3): An improved simulation model for rice in drought and nitrogen-deficient environments

**DOI:** 10.1016/j.agrformet.2017.02.025

**Published:** 2017-05-01

**Authors:** Tao Li, Olivyn Angeles, Manuel Marcaida, Emmali Manalo, Mervin Pogs Manalili, Ando Radanielson, Samarendu Mohanty

**Affiliations:** International Rice Research Institute, Los Baños, Philippines

**Keywords:** ORYZA2000, ORYZA (v3), Rice ecosystem, Drought stresses, Nitrogen deficiency, Model improvement

## Abstract

The worldwide usage of and increasing citations for ORYZA2000 has established it as a robust and reliable ecophysiological model for predicting the growth and yield of rice in an irrigated lowland ecosystem. Because of its focus on irrigated lowlands, its computation ability is limited to the representation of the effects of the highly dynamic environments of upland, rainfed, and aerobic ecosystems on rice growth and yield. Additional modules and routines to quantify daily variations in soil temperature, carbon, nitrogen, and environmental stresses were then developed and integrated into ORYZA2000 to capture their effects on primary production, assimilate allocation, root growth, and water and nitrogen uptake. The newest version has been renamed “ORYZA version 3 (v3)”. Case studies have shown that the root mean square errors (RMSE) between simulated and measured values for total biomass and yields ranged from 11.2% to 16.6% across experiments in non-drought and drought and/or nitrogen-deficient environments. ORYZA (v3) showed a significant reduction of the RMSE by at least 20%, thereby improving the model’s capability to represent values measured under extreme conditions. It has also been significantly improved in representing the dynamics of soil water and crop leaf nitrogen contents. With an enhanced capability to simulate rice growth and development and predict yield in non-stressed, water-stressed and nitrogen-stressed environments, ORYZA (v3) is a reliable successor of ORYZA2000.

## Highlights

• Improvements in ORYZA (v3) were identified and compared to ORYZA2000.
• Soil carbon, nitrogen, and temperature dynamic modules were developed.
• Algorithms for the effects of environmental stresses on rice growth were improved.
• Case studies confirmed successful improvement of the model.

## Nomenclature

IRRI: International rice research institute
*T_a_*: Air temperature of the soil layer (°C)
*K*: Thermal conductance of the soil layer (J s^−1^ m^−1^ k^−1^)
*z*: Depth of the soil layer (m)
*H*: Heat storage capacity of the soil layer (J m^−3^ k^−1^)
*i*: Specific soil layer
*Cl*: Soil clay content in a given soil layer (g g^−1^)
*Sa*: Sand content in a given soil layer (g g^−1^)
*θ*: Water content in a given soil layer (m^3^ m^−3^)
C: Carbon content (kg C ha^−1^)
N: Nitrogen content (kg C ha^−1^)
*BD*: Bulk density (Mg m^−3^)
*pH*: Soil pH
*D_max_*: Maximum rooting depth which is a genetic character of a given cultivar (m)
*T*: Temperature of the soil layer (°C)
SWC: Soil water content (m^3^ m^−3^)
WCWP: Soil water content at wilting point (m^3^ m^−3^)
WCST: Soil water content at saturation (m^3^ m^−3^)
WCFC: Soil water content at field capacity (m^3^ m^−3^)
DTF: Drought tolerance factor
WS: Wet season
DS: Dry season
SN: Nitrogen deficiency stress
LAI: Leaf area index
WME: Water management experiment
AWD: Alternate wet and dry water management
NFM: Nitrogen fertilizer management with different nitrogen doses and splits
ARE: Aerobic rice experiment
MVD: Multiple variety drought experiment
S_w_: Drought stress index
PAU: Punjab agricultural university
CF: Continuously flooded
DAE: Days after emergence (day)
W1: Water management practice 1: irrigated every three days between emergence and flowering
W2: Water management practice 2: three irrigations in every three days starting at emergence, and one additional irrigation between panicle initiation and flowering
N: Nitrogen amount (kg N ha^−1^)
Y: Year
α: Intercept of linear regression
*β*: Slope of linear regression
*P(t)*: Student’s *t*-test
*r^2^*: Correlation coefficient
*RMSEn*: Normalized root mean square error
*M_eff_*: Model efficiency
*i*: I^th^ data pair
*n*: Number of x and y data pairs
$\bar{X}$: Average of all measurements of a variable in an experiment
WAGT: Total above-ground biomass (kg ha^−1^)
WSO: Total panicle biomass (kg ha^−1^)
WST: Total stem biomass (kg ha^−1^)
WLVG: Total green leaf biomass (kg ha^−1^)
Leaf_N: Green leaf nitrogen content (g N m^−2^ leaf)
STRASA: Stress-tolerant rice for Africa and South Asia

## 1 Introduction

Rice is the staple food for more than half of the world’s population. Between the early 1960s and the early 1990s, global rice consumption more than doubled, from 150 million tons to 350 million tons, due to a combination of rising per capita consumption and population growth (Mohanty, 2013). Since then, per capita consumption growth has slowed due to the diversification of diets, primarily in Asian countries, but total consumption continues to increase due to population growth and a rapid increase in consumption in Africa. In the past decade (2006–2016), total global rice consumption has increased by 57 million tons, an increase of nearly 14%. According to Seck et al. (2012), total global consumption is projected to increase by another 116 million tons (milled equivalent) in the next 25 years.

The currently harvested rice area of 160 million hectares is at an all-time high. Since it is unlikely for the rice area to expand further in the future, productivity will have to increase to meet the growing demand and keep rice affordable for millions of poor people. Of the various ecosystems in which rice is grown, rainfed areas, which account for approximately 38% of the global rice-producing area and contribute only 23% to total rice production, could be targeted for productivity improvement (GRiSP, 2013). The productivity of rainfed rice ranges from 1.5 to 2.5 t ha^−1^ (Raman et al., 2012) and is limited by biotic and abiotic stresses. Of abiotic stresses, drought is the most serious and can cause as much as a 40% loss in annual production and a 58% loss of income in South and Southeast Asia (IRRI, 2009, Pandey and Bhandari, 2007, Pandey and Bhandari, 2006).

The effects of climate change, particularly the continuous increase in temperature and the increase in the frequency, duration, and severity of water shortages (Spinoni et al., 2014) have become increasingly serious constraints that will limit rice production in the future (Wassmann et al., 2009a, Wassmann et al., 2009b). Understanding the variation and the effects of improved rice varieties and agronomic management on rice productivity in drought- and nutrient deficiency-prone environments (i.e., non-flooded uplands, rainfed, and aerobic rice ecosystems) is critical for developing adaptive technologies for these environments.

Crop modeling technology provides time- and cost-effective means to extrapolate findings from a limited number of field and laboratory studies to larger spatial and temporal scales for the development of adaptation technology. In lowland rice systems, a large number of application studies have shown that ORYZA2000 is a robust model that provides reliable predictions for rice growth and yield in irrigated systems. ORYZA2000 is a crop model that integrates ORYZA1 (Kropff et al., 1994), ORYZA_N (Drenth et al., 1994), and ORYZA_W (Wopereis et al., 1996), and it has been continuously used to identify better rice crop management options, assess the effects of climate change, and assist in the dissemination of technology and the release of rice varieties and rice breeding for targeted environments (Aggarwal and Mall, 2002, Artacho et al., 2011, Das et al., 2007, Feng et al., 2007, Li et al., 2016, Li et al., 2013, Sudhir-Yadav et al., 2012, Sudhir-Yadav et al., 2011, Vaghefi et al., 2011). The evolution of ORYZA2000 (Bouman et al., 2001) to ORYZA (v3) includes a long list of progeny, which has been continuously developed and updated by the International Rice Research Institute (IRRI) in Los Banos, Philippines for the last 25 years. ORYZA (v3), which is the latest version that has been released, is the result of a continued effort to improve the model regarding simulating the growth and yield of rainfed, upland, and aerobic rice as affected by drought stress and an associated nitrogen deficiency. This paper introduces ORYZA (v3), emphasizing its modifications, new routines, and modules, using four case studies to verify the improved capabilities of ORYZA (v3) for the simulation of the growth and development of flooded and non-flooded rice.

## 2 The development of ORYZA (v3) from ORYZA2000

Rainfed, upland and aerobic rice ecosystems have complex dynamics due to higher vulnerability to climate and abiotic stresses, while irrigated lowland ecosystems have less fluctuations in soil moisture and temperature due to the constant presence of standing water. To represent the effects of the soil, air and water environmental variability on rice growth, development, and yield, ORYZA (v3) was developed by modifying some computation algorithms of ORYZA2000 and integrating additional modules and routines.

### 2.1 Changes in the model structure

**Fig. 1:**
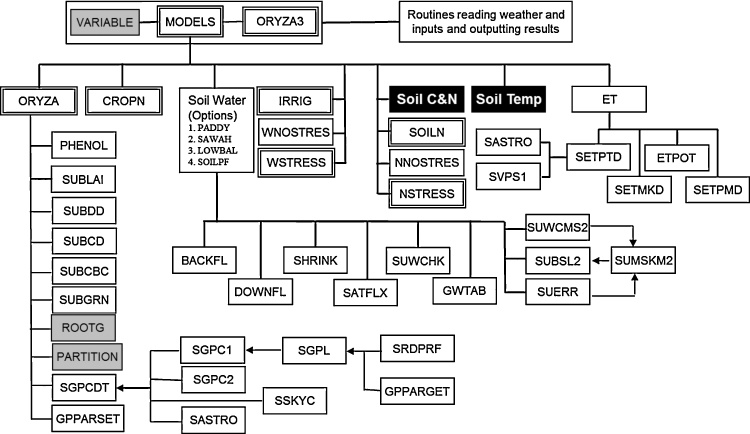
The model structure of ORYZA (v3), which has an improved capability to simulate rice growth and yield for lowland, as well as upland, rainfed, and aerobic rice ecosystems. Modified routines are shown in double-framed boxes, while new modules and routines are shown in black and gray boxes, respectively. Lines connecting the boxes indicate the two-way flow of information and/or mass exchange, while single-headed arrows indicate unidirectional mass and/or information flow.

The older versions of ORYZA, ORYZA2000 v1 to v2.13, provide reliable simulations of rice growth in lowland ecosystems and changes (Fig. 1) were implemented on the model to develop an upgraded version. New modules (black boxes) and additional routines (gray boxes) were developed and integrated, while old routines (double-frame boxes) were modified to achieve additional and/or alternative functions in ORYZA (v3).

### 2.2 The new modules

#### 2.2.1 Soil temperature dynamics

In non-flooded rice fields, the seasonal and diurnal amplitude of the soil temperature is much larger due to the absence of the heat buffering capacity that standing water provides in flooded lowland fields. Greater soil temperature fluctuation strongly affects the growth and distribution of the roots in the soil profile, the uptake of water and nutrients, and the transformation of carbon and nitrogen in the soil.

**Fig. 2:**
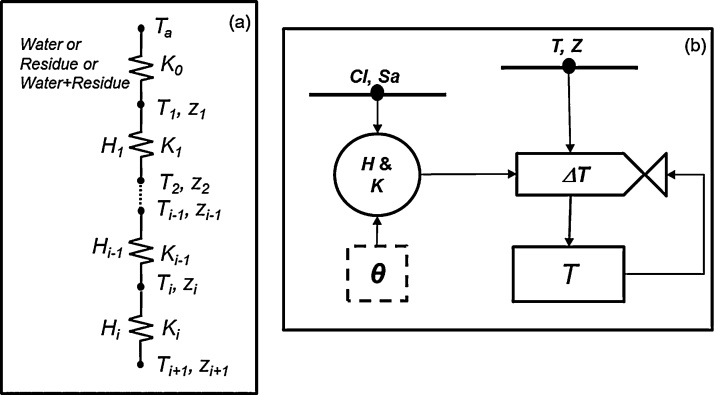
Diagram of soil temperature and heat flux computation in a soil profile (a) and a flowchart for the calculation of the temperature of each soil layer (b). *T_a_* is the air temperature; *T*, *K*, *z,* and *H* are the temperature, thermal conductance, depth, and heat storage capacity of a soil layer, respectively; and *i* indicates the specific soil layer. *Cl*, *Sa*, and *θ* are the soil clay, sand, and water content, respectively, in a given soil layer.

To represent the effects of the soil temperature on root growth, water and nutrient uptake, and the nutrient cycle, a soil temperature module was developed to calculate the daily soil temperature in soil layers from the surface to the lower boundary using Fourier Law (Fig. 2) (Supplementary Section 1.1 for the equations and computation process).

The heat capacity (*H_0_*) and thermal conductance (*K_0_*) of the surface layer vary if the surface layer is either inundated with standing water, spread with crop residue, or a mixture of both. The soil moisture content, surface water depth (mm), air temperature (°C), and the type and amount of crop residue (kg ha^−1^), are the inputs needed for the soil temperature module. The daily average soil temperature in each layer is the output and is used as the input for the soil carbon and nitrogen dynamics module as well as an environmental factor that affects root growth.

#### 2.2.2 Soil carbon and nitrogen dynamics

**Fig. 3:**
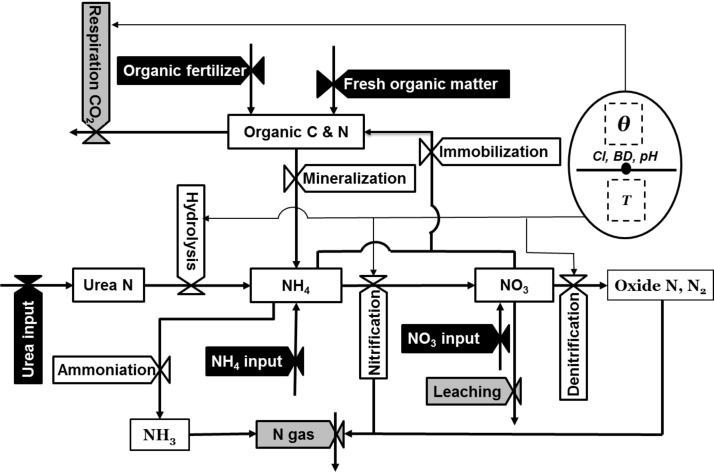
Flowchart for the soil carbon and nitrogen dynamics module. Solid boxes are state variables; white, black, and gray valves indicate transformation and input and output rates, respectively. The output rates indicate the rate of decrease in mass leaving the boundary of the module. Dashed boxes are state variables taken from other modules and used as inputs to a succeeding module. The components in the ovals including the external input data (solid line with a dot) and the state variables from/to another module (dashed boxes) are the information needed for rate computations. The thick solid lines represent the mass flow while the thin solid lines illustrate the information flow. *BD* and *pH* are the soil bulk density and the soil pH, respectively.

Soil carbon and nitrogen transformations are strongly affected by environmental factors such as soil moisture and temperature. In non-flooded rice fields, fluctuations in these factors result in unstable nutrient transformations. The versions of ORYZA2000 do not compute soil carbon and nitrogen transformation, and the SOILN routine uses a predetermined value for nitrogen mineralization. In ORYZA (v3), a soil carbon and nitrogen dynamics module adopted from Li et al. (2013) was developed to quantify (a) the amount of mineral nitrogen in the soil, (b) the amount of nitrogen lost through leaching, volatilization, and denitrification, and (c) changes in the soil organic carbon and nitrogen content (Fig. 3). The processes of carbon decomposition, nitrogen mineralization and immobilization, urea hydrolysis, the nitrification of ammonium, the denitrification of nitrate, the volatilization of ammonia, and nitrogen deposition from the atmosphere are calculated by the module (Supplementary section 1.2 for the detailed description and computation equations).

The module considers the fresh organic matter and organic fertilizer (kg C and N ha^−1^) as external sources of organic carbon and nitrogen. The fresh organic matter could either come from crop residues or organic matter from biological fixation possibly in a lowland field. The nitrogen fertilizer and deposition of atmospheric nitrogen (wet or dry) are additional sources of mineral nitrogen (Fig. 3, black valves). This module also estimates carbon dioxide and nitrous oxide emissions (Fig. 3, gray valves) and assumes that the movement of mineral nitrogen is concurrent with the water movement between the soil layers (Supplementary section 1.2). No independent exchange of soil organic carbon and nitrogen between layers is assumed. Hence, this module does not simulate soluble organic carbon.

### 2.3 New and modified routines

#### 2.3.1 Rice root growth routine

Soil moisture, temperature, and nutrients vary among soil profile layers. Root growth, including its distribution among layers in the soil profile, differs between water-limited and non-limited conditions (Mitchell et al., 2012). In previous versions of ORYZA2000, roots grow downward at a constant rate and root biomass is equivalently distributed in the rooted soil profile. The response to differences in temperature, moisture, and nutrient status of the soil layers was not included.

**Fig. 4:**
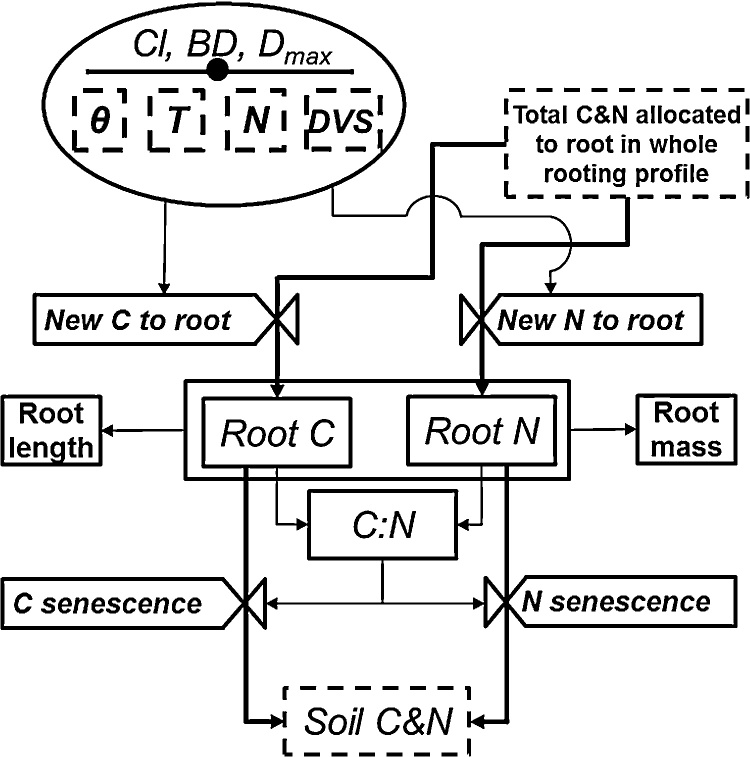
A flow chart illustrating the computation of root growth in each soil layer. *D_max_* is the maximum rooting depth, which is a genetic characteristic of a given cultivar. *Cl* and *BD* are the soil clay content and bulk density, respectively. *θ* is the water content; *T* is the temperature; N is the N content; DVS is the specific developmental stage, and *i* indicates a specific soil layer. White valves indicate transformation rates. Dashed boxes are state variables taken from other modules and used as inputs for this module. The components in the circles including the external input data (solid line with a dot) and state variables from/to another module (dashed boxes) are information needed for rate computations. Thick solid lines represent the mass flow while thin solid lines illustrate information flow.

This routine was modified to simulate root growth and the distribution of the root biomass including the carbon and nitrogen content of the soil layers considering the variability in the soil texture, temperature, moisture, and nitrogen content (Fig. 4). Root senescence is determined by root nitrogen to meet the optimal root carbon to nitrogen ratio, which is a genetic characteristic of a given cultivar (Yin et al., 2009) (Supplementary section 1.3 for detailed computation and equations).

#### 2.3.2 Water uptake and drought stress quantification

In the versions of ORYZA2000, the total amount of water extracted by the plant is equally distributed in all rooted soil layers regardless of the root mass or the length density and the soil moisture in the root zone. When the total extractable water is less than the potential transpiration, the resultant water shortage is compensated from the topmost to the lowest rooted soil layer until the soil moisture content reaches the wilting point. For any day during crop growth when the total water taken up is less than the potential transpiration, the drought stress index (*S_w_*) is calculated based on the soil water potential.

In ORYZA (v3), considering the variation in soil moisture, temperature, and nutrients in the soil profile of non-flooded rice fields, the water uptake in a given layer is determined based on two factors: 1) the ratio of root mass in the layer to the total mass in the root zone, and 2) the ratio of extractable water in the layer to the total extractable water in the root zone (Supplementary section 1.4.1).

The amount of extractable soil water is the portion of the available soil water given by the difference between the soil water content (SWC) at any given time and the soil water content at the wilting point (WCWP). The maximum available soil water is the difference between the soil water content at saturation (WCST) and WCWP. The amount of water between WCST and the soil water content at field capacity (WCFC) is also counted as available soil water simply because rice is a hydrophilic plant. The amount of extractable water is the same as the maximum available water when the SWC is equal to WCST, and then decreases to the portion of available water as the SWC approaches the WCWP (Figs. S1 and S2). The decreasing ratio between available and extractable water is determined by the drought tolerance factor (DTF) (Eq. S69). The DTF is defined as the ratio of the maximum available water in the soil to the extractable water at which plant transpiration starts to significantly decrease (Fig. S1).

Drought stress is quantified by an exponential function of the ratio of the water uptake to the water demand (i.e., the potential transpiration) (Eq. S72) (Li et al., 2009, Ronda et al., 2001). A drought stress index (*S_w_*) is used to scale down the potential photosynthesis to actual photosynthesis (Fig. S3), thus adjusting the assimilate allocation among the plant organs in drought stress conditions (Supplementary section 1.5). The DTF has a value ranging from 1 to 10 and limits the rate of this reduction in photosynthesis depending on the varietal differences in drought tolerance (Heinemann et al., 2011, Pereira et al., 2012). Photosynthesis linearly decreases with the decrease in the soil water content for a non-drought tolerant rice variety (DTF = 1) and would not be significantly lower under a mild drought stress for a drought-tolerant variety (DTF > 1.0) (Fig. S3).

#### 2.3.3 Nitrogen uptake and stress quantification

**Fig. 5:**
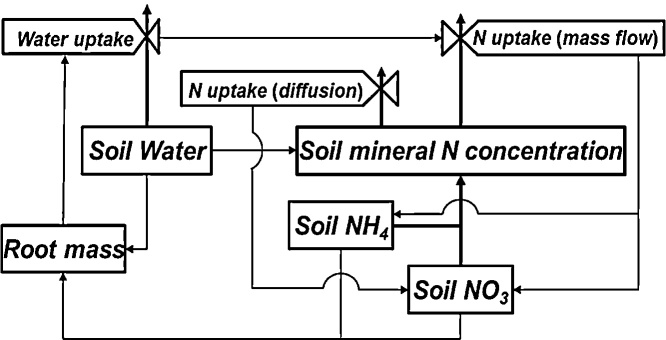
Illustration of the coupling scheme of the water and the nitrogen uptake. Solid boxes represent state variables while the tied valves represent rate variables. The thick line arrows indicate the mass flow while the thin line arrows represent the information input for computing the state and rate variables.

The versions of ORYZA2000 assumed that all mineral nitrogen in the soil is available as needed and that the actual nitrogen uptake is the lower value of the maximum uptake capability of a given cultivar and the total available soil mineral nitrogen. It also assumed that the soil moisture and the root distribution in the soil profile did not affect the nitrogen uptake. In ORYZA (v3), the nitrogen uptake is coupled with the water uptake (Fig. 5; Supplementary section 1.4.3).

Nitrogen uptake has two types of paths: mass flow and diffusion, which are directly and indirectly affected by the water uptake, respectively. The amount of soil water and nitrogen affect the root growth and mass distribution in the soil profile. The root mass is one of the major factors that governs the water uptake. With these interactions, root mass, soil water content, and nitrogen concentration form a closed coupling cycle. An increase in the soil water content dilutes the nitrogen concentration, which reduces the amount of nitrogen taken up per unit of water absorbed. An increase in diffusive uptake partially compensates for the reduction in the uptake of N via mass flow. In this case, a decreasing soil water content increases the nitrogen concentration and the diffusive nitrogen uptake. A severe drought will significantly decrease the total amount of water taken up as well as the mass flow of nitrogen, resulting in both drought and nitrogen deficiency stresses. Either or both drought stress and low soil fertility could result in a nitrogen deficiency (Gonzalez-Dugo et al., 2010).

Nitrogen deficiency stress (SN) is quantified as the ratio of the actual nitrogen uptake to the nitrogen demand (Supplementary section 1.4.4), which decreases the rates of photosynthesis and transpiration. The reduction in the transpiration rate induced by SN or drought stress results in a reduction in the potential photosynthesis. The greater stress of SN or drought stress is the factor that determines the reduction in the transpiration and photosynthesis rates and adjusts the assimilate allocation among the plant organs (Supplementary section 1.5).

#### 2.3.4 Assimilate allocation among plant organs under drought and nitrogen deficiency stress

Biomass allocation to plant organs is modified by drought stress, nitrogen deficiency, and competition for light on each growth day using the computation algorithms modified from Friedlingstein et al. (1999) and Li et al. (2009) (Supplementary section 1.5). Drought and nitrogen stress favor the translocation of more assimilate to the roots rather than to the shoots (Fig. S6). More roots allow plants to explore a larger soil volume for water and nitrogen. Of the above-ground organs, more assimilate is allocated to the leaves and storage organs rather than to the stem, which enables the plant to have larger leaf area to maintain productivity and grain yield. Competition for light results in more assimilates translocated to the stem and storage organs, which produces a larger spatial volume to capture more solar radiation or to reduce the size of its inefficient forage to maintain the size of storage. Ultimately, the computation algorithm gives the highest priority to storage organs under abiotic stress conditions (Fig. S6).

## 3 Evaluation of ORYZA (v3)

The performance of ORYZA (v3) was evaluated using four experiments under different water and nitrogen management practices in different rice ecosystems (Table 1). The objective was to assess the ability of and verify the improved accuracy of the model to simulate rice production under water- and/or nitrogen-limited conditions and in aerobic rice after integrating the modifications into the previous version (ORYZA2000).

### 3.1 Experiments

#### 3.1.1 Water-limitation experiment under AWD management

The data used to assess the model’s ability to simulate rice production under various drought stress conditions were obtained from field experiments carried out at the research station at Punjab Agricultural University (PAU), in Ludhiana, India (30°54′ N, 75°98′ E, elevation 247 m) in 2008 and 2009 (Table 1, Table 2). Detailed information about the experiment was presented in Sudhir-Yadav et al. (2011). The water management schemes were daily irrigation and alternate wetting and drying (AWD) with threshold soil water potentials of 20, 40, and 70 kPa at an 18–20 cm depth. For AWD, approximately 50 mm of irrigation water was applied to the field when soil water potential reached the threshold. The AWD management regime was implemented 15 days after transplanting. In this experiment, rice was grown in environments with various drought stress levels (from no drought to severe drought). The plant organ biomass and the leaf area index (LAI) were measured six times during the growth season in 2008 and 2009 (Table 2). The soil water potential in each treatment was monitored using a tensiometer (IRROMETER Company Inc., Riverside, CA, USA).

#### 3.1.2 Nitrogen management experiment

A nitrogen fertilizer management experiment (NFM) was carried out in the dry and wet seasons from 1992 to 1993 at a farm at IRRI (14°13′N, 121°15′E, elevation 23 m), Los Banos, Philippines. The observed data on time-series changes of the LAI and organ biomass were used to evaluate the capability of the model to simulate rice growth in the presence of different levels of nitrogen with different fertilizer amounts and splits in a continuously flooded lowland (Table 3). The details of this experiment were described in Li et al. (2009).

#### 3.1.3 Aerobic rice experiment

The aerobic rice field experiment (ARE) was carried out from April to October 2003 and 2004 at the Changping Experiment Station (40°2′N, 116°10′E; elevation 43 m) at the China Agricultural University near Beijing (Yang et al., 2005) (Table 1). This experiment involved one rice cultivar, HanDao297, and two water and fertilizer management schemes. The observed changes in the LAI and organ biomass with time were used to evaluate the model’s capability to represent rice growth in an environment in which water and nitrogen interactions occurred (Table 4).

#### 3.1.4 Drought stress experiment with multiple varieties

The drought stress experiment with multiple varieties (MVD) was conducted at the IRRI Experiment Station during the dry season of 2011 (S1) and 2012 (S2). The goal of the experiment was to evaluate the tolerance of the varieties to drought stress in two contrasting environments (full irrigation and a severe drought with a soil moisture potential down to −500 kPa, Marcaida et al., 2014). The data collected for seven varieties under fully irrigated and drought stress (i.e., rainfed during the reproductive stage of the rice) environments during two growing seasons were used to evaluate ORYZA (v3). For each variety, one of the two seasons was randomly selected for calibration (codes in bold) while the remaining one was used for validation (Table 5).

### 3.2 Model simulation and evaluation

Following the standard protocol used for the evaluation of the ORYZA2000 model (Li et al., 2009), each experimental dataset was split into two subsets for model calibration and validation (Table 2, Table 3, Table 4, Table 5). Simulations for both calibration and validation were organized according to the crop management in the corresponding field experiment. The cultivars in each experiment were parameterized through the process of calibration using data on crop phenology, the measured LAI value, and the organ biomass. After the calibration, the performance of the model was evaluated using the validation dataset. The calibration and validation process were conducted for both versions to verify the improvements made in ORYZA (v3).

For each measured variable (i.e., LAI, organ biomass, leaf N content, grain yield, and soil water potential), the simulated data on specific dates of measurements were extracted from the daily simulation outputs. The measured and simulated data were represented as X, Y data pairs. The statistical parameters (i.e., α and *β* for the intercept and slope, respectively) of the linear regression between X and Y were then calculated, and Student’s *t*-test under the assumption of unequal means (*P(t)*) was carried out as well as a correlation analysis (*r^2^*, Eq. (1)). The normalized root mean square error (*RMSE_n_*, Eq. (2)) was also used to quantify the relative error (i.e., root mean square error, RMSE) of the simulated mean. The model efficiency (*M_eff_*, Eq. (3)), which is highly sensitive to extreme values, represents the total variation of measurements explained by the model.

(1) $$r^{2}=\frac{{\left(\sum_{i=1}^{n}\left(X_{i}Y_{i}\right)-\sum_{i=1}^{n}X_{i}\sum_{i=1}^{n}Y_{i}\right)}^{2}}{(\sum_{i=1}^{n}{\left(X_{i}\right)}^{2}-{\left(\sum_{i=1}^{n}X_{i}\right)}^{2})\left(\sum_{i=1}^{n}{\left(Y_{i}\right)}^{2}-{\left(\sum_{i=1}^{n}Y_{i}\right)}^{2}\right)}$$

(2) $$RMSE_{n}=\frac{\sqrt{\frac{\sum_{i=1}^{n}{\left(Y_{i}-X_{i}\right)}^{2}}{n}}}{\bar{X}}$$

(3) $$M_{eff}=1.0-\frac{\sum_{i=1}^{n}{\left(X_{i}-Y_{i}\right)}^{2}}{\sum_{i=1}^{n}{\left(X_{i}-\bar{X}\right)}^{2}}$$

In Equs. (1)–(3), *n* is the number of X, Y data pairs, while *i* denotes the *i^th^* data pair, and $\bar{X}$ is the average of all measurements of a variable in an experiment.

The statistical analyses were implemented for the calibration and validation datasets separately so that the confidence level of the model for representing the measurements could be clearly identified. Ideally, the model’s performance would be considered good when the values of *β*, *r^2^*, and *M_eff_* were close to 1.0, α and *RMSE_n_* were close to zero, and *P(t)* was larger than 0.05.

## 4 Results and discussion

### 4.1 Calibration and validation for biomass and yield

Case studies used for model evaluation covered different water environments for rice production, from fully irrigated and intermediate drought stress conditions in WME and ARE to severe drought stress in MVD. The data also covered nitrogen environments varying from zero to excessive amounts of nitrogen application with 1–4 splits in the NFM. Using the calibration datasets (Table 2, Table 3, Table 4, Table 5), the cultivars and soil parameters in the WME, NFM, ARE and MVD were estimated to ensure that simulated and observed values are within acceptable fitness as illustrated by the corresponding statistical variables namely, α, *β*, *r^2^*, *P(t), RMSEn*, and *M_eff_* (Table 6 and S1). The calibration exercise successfully enabled ORYZA (v3) and ORYZA2000 (v2.13) to represent the dynamics of biomass accumulation in the different production environments (Table 6).

**Fig. 6:**
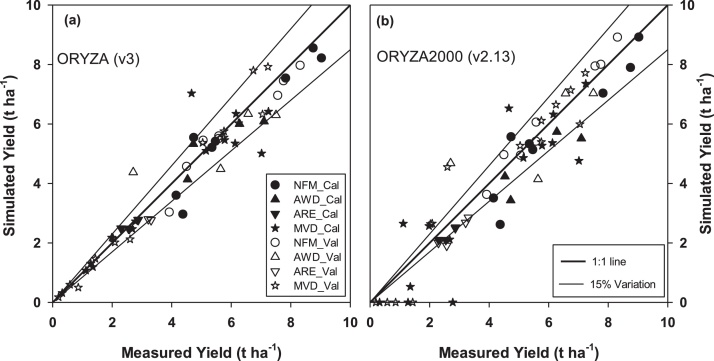
The measured and simulated grain yields used during calibration (solid symbols) and validation (open symbols). Panel **a** presents the results generated from ORYZA (v3) and panel **b** from ORYZA2000 (v2.13). The thick line is the 1:1 line, and the thin lines define the 15% variation in the range of measurements. The Cal and Val in the legend codes indicate the calibration and validation datasets, respectively, while the NFM, AWD, ARE and MVD are the codes for the four study cases (Table 1).

**Fig. 7:**
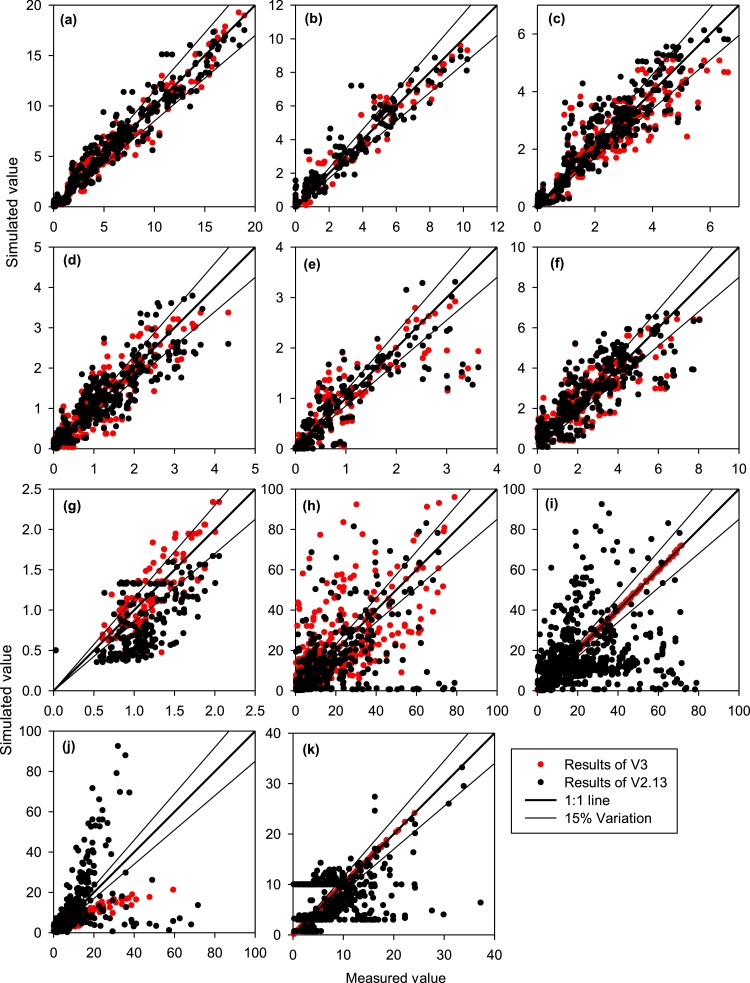
The observed plant organ biomass (Mg ha^−1^), leaf area index, leaf N content (g N m^−2^ leaf), and soil water potential (kPa) together with the values simulated by ORYZA (v3) (red dots) and ORYZA2000 (v2.13) (black dots). Panels **a** to **e** are total above-ground biomass, panicle, stem, green leaf and dead leaf biomass. Panels **h** to **k** are the soil water potential for second to fifth soil layers below the surface, and panels **f** and **g** are the leaf area index and nitrogen content, respectively. (For interpretation of the references to colour in this figure legend, the reader is referred to the web version of this article.)

With the validation datasets, the statistical parameters describing the agreement of the simulated to observed values were in the same range as the calibration datasets. ORYZA (v3) was able to represent 93% (r^2^ ≥ 0.93) of the measured biomass of the plant organs across different rice production environments, and it was effective in representing the amplitude of variation from small to large amounts of biomass (*M_eff_* ≥ 0.85) (Table S1). It also had similar prediction accuracy in different production environments, varying from full water and nitrogen supply to severely water- and nitrogen-stressed (Fig. 6, Fig. 7), while the earlier version was highly reliable for the good water and nitrogen production environment but less reliable for stressed environments (Li et al., 2009).

ORYZA (v3) had also demonstrated an improved capability to represent growth dynamic production environments with strong water and nitrogen interactions as are often observed in ARE and MVD experiments (Table 6; Fig. 6, Fig. 7, S4 and S8). The RMSE_n_ between predicted and measured grain yields was less than 15% of the measured average in ORYZA (v3) and was significantly lower than that of ORYZA2000 (v2.13) at 21% (Table 6). The measured yields in different crop management and production environments were represented by the model for more than 95% (*r^2^* > 0.95) (Table 6 and S1). ORYZA (v3) was also able to efficiently (*M_eff >_* 0.87) identify the wide range of yields, from the lowest yields in aerobic and severe drought conditions to the highest under full water and nitrogen supply, which was better than the previous version (*M_eff_* ≥ 0.90 vs. 0.84; Table 6 and S1, Fig. 6). The prediction uncertainties on biomass and yield were 20% lower in ORYZA (v3) (Table 6, Figs. 7, S7 and S8). Apparently, ORYZA2000 (v2.13) overestimated the impact of severe drought (Fig. 6b) because of the less accurate estimations of soil water potential (Fig. 7h–k).

### 4.2 Calibration and validation for leaf area and nitrogen content dynamics

Both ORYZA (v3) and ORYZA2000 (v2.13) have less accuracy on the predictions for leaf nitrogen content than those for plant biomass and yield (Table 6). ORYZA (v3) predicted leaf nitrogen (N) contents with the RMSE_n_ normally at approximately 30% of the measured mean. It was able to capture 80% of the variation of the observed leaf N contents. It was effective enough to capture extremely high or low leaf N contents for both calibration and validation datasets (Table S1). The large biases that occurred in the initialized leaf N contents (Fig. S9) considerably contributed to the lower agreement between the simulated and observed values, thus reducing the accuracy of the simulations as illustrated by the lower statistical parameters (Table S1). The simulations of ORYZA (v3) presented a good fit with the leaf N dynamics within seasons under different nitrogen management with or without water limitations. The model was able to predict the low leaf N content with the higher nitrogen application in the aerobic experiment as well as the high leaf N content in the low nitrogen fertilizer application in fully irrigated environments (Fig. S9, Tables 2 and 4), which confirmed the capability of ORYZA (v3) to represent the interaction of water and nitrogen. With the reduction of the uncertainties on leaf N contents by 30% from the prediction uncertainties of ORYZA2000 (v2.13) (Table 6, Figs. 6 and S9), ORYZA (v3) can provide reliable results that can be used for improving nitrogen management in different rice ecosystems.

### 4.3 Soil water potential/content dynamics

The simulation accuracy of ORYZA (v3) on soil water potential was higher for deeper soil layers (≥10 cm depth) than for upper soil layers (≤10 cm depth) (Table S1). Soil water potential in the upper layers was strongly influenced by field management and daily weather conditions. The simulation accuracy in the soil water potential in deeper layers was relatively better as these layers were normally monitored for AWD water management, as observed in the WME and ARE experiments. The prediction uncertainty of ORYZA (v3) is approximately 70% higher than of that of ORYZA2000 (v2.13) within the soil profile, although the improvement was not significant for the upper soil layers (Table 6, Figs. 7 and S10). ORYZA (v3) is better than previous versions for water management optimization.

## 5 Conclusions

ORYZA (v3) is the successor of ORYZA2000, and this rice model has a stronger capability to simulate rice growth and development dynamics. ORYZA (v3) has a wider applicability domain regarding rice production environments than ORYZA2000 (v2.13) and has achieved a higher confidence level for the prediction of rice growth and yield (Tables 6 and S1, Figs. S1 to S8). The implementation of additional modules for soil nutrient dynamics, root growth, and soil temperature allow more accurate simulations of yield, plant organ biomass, leaf area, and leaf N content as well as the dynamics of the soil water potential. The high accuracy of ORYZA (v3) for various production environments ensures its reliable application for rice crop management and yield prediction from the more uniform field scale to a regional scale, at which the rice production system and environmental conditions vary tremendously.

ORYZA (v3) simulates phenology with parameters that vary with genotype, environment, and management, which is the same weakness as in earlier versions. For the model to be used for long-term studies or at larger spatial scales, the input parameters that control phenology should be the average values obtained from as many environments and management conditions as possible. This weakness, however, will be addressed in the next version of the ORYZA rice model. Cultivar parameters, except those for phenology, have been implemented as genetic parameters in ORYZA (v3). Consequently, intensive calibration is required to achieve an accurate prediction from the model. The intensive calibration is now managed more efficiently using the auto-calibration tool developed with the new version.

## Figures and Tables

**Table 1:** Descriptive information for the experiments used in the evaluation of the ORYZA2000 and ORYZA (v3).

Dataset^a^		WME	NFM	ARE	MVD
Location		Ludhiana, Punjab, India	Los Banos, Philippines	Changping, Beijing, China	Los Banos, Philippines
Experimental year		2008, 2009	1992 wet season (WS) & 1993 dry season (DS)	2003, 2004	2011 & 2012 dry seasons (DS)

Seasonal	Average temp. range (°C)	24.9–33.7	21.9–30.4 (DS), 24.0–31.3 (WS)	20.0–29.9	22.7–28.7 in 2011 & 23.4–29.8 in 2012
	Total rad (Mj)	1175.9	1291.3 (DS), 901.4 (WS)	454.0	1398.5 in 2011 & 1480.6 in 2012
	Total precip. (mm)	564.1	62.7 (DS), 976.5 (WS)	596.1	217.0 in 2011 & 309.1 in 2012
	Total ET (mm)	603.5	544.7 (DS), 479.5 (WS)	558.4	477.7 in 2011 & 440.4 in 2012

Cultivar/Genotype		PAU201/Indica	IR72/Indica	Handao297/Japonica	7 varieties
Seedling age (days)		23 in 2008, 24 in 2009	12	Direct-seeding	29
Planting density (hills m^−2^, plants hill^−1^)		33, 2	25, 5	260 plants m^−2^	25, 5
Water management		Fully irrigated, and AWD with three different thresholds	Fully irrigated	Flash flooding as needed	Fully irrigated or rainfed in reproduction stage
N fertilizer management		120 in three equal splits	0 to 225 kg N ha^−1^ with six types of splits	225 and 300 kg N ha^−1^ in 3 equal splits	160 kg N ha^−1^ in 5 splits (30:30:30:30:40)
Measurements		LAI & organ biomass	LAI & organ biomass	LAI & organ biomass	LAI & organ biomass

a WME – water management experiment; NFM- nitrogen fertilizer management experiment; ARE – aerobic rice experiment; MVD- multiple varieties with drought stress experiment; LAI – leaf area index.

**Table 2:** The water management experiment (WME) with continuously flooded (CF) and alternate wet and dry (AWD) management with three different thresholds used to evaluate ORYZA (v3). Treatment codes in bold indicate the treatments used for model calibration while all the other treatments were for model validation.

Experiment year	Water management or AWD threshold for soil water tension at 15 cm depth of soil			
	Continuously flooded (CF)	20 kPa (W1)	40 kPa (W2)	70 kPa (W3)
2008	**CF-08**	W1-08	**W2-08**	W3-08
2009	CF-09	**W1-09**	W2-09	**W3-09**

**Table 3:** The nitrogen fertilizer management experiment (NFM) with different amounts of total nitrogen (N) rates (kg N ha^−1^), splits applied in different number of days after emergence (DAE), and the amount at each split (kg N ha^−1^). The treatments with codes in bold were used for calibration and the remaining treatments were used for validation in the wet season (WS) in 1992 or dry season (DS) in 1993.

Experiment in the wet season of 1992						Experiment in the dry of 1993					
DAE for split	12	34	56	80	Total N applied	DAE for split	12	34	54	76	Total N applied
Treatment code	N applied at each split					Treatment code	N applied at each split				
**WSN1**	0	0	0	30	30	**DSN1**	0	0	0	0	0
WSN2	80	0	0	30	110	DSN2	0	0	0	45	45
**WSN3**	40	40	0	30	110	**DSN3**	60	60	60	0	180
WSN4	27	27	27	30	111	**DSN4**	60	60	60	45	225
WSN5	0	0	0	0	0	DSN5	60	60	0	0	120
**WSN6**	80	0	0	0	80	**DSN6**	60	60	0	45	165
WSN7	40	40	0	0	80	DSN7	0	60	60	0	120
**WSN8**	27	27	27	0	81	DSN8	0	60	60	45	165

**Table 4:** The aerobic rice experiments (ARE) with two water management practices and two nitrogen fertilizer application rates. The data from the treatments highlighted in bold were used for calibration, and the remaining treatments were for validation.

N applied		N1: 225 kg N ha^−1^		N2: 300 kg N ha^−1^	
Year		Y1: 2003	Y2: 2004	Y1: 2003	Y2: 2004
Water management	W1: Irrigated every 6–24 days between emergence and flowering to ensure the soil water potential at 15 cm depth was ≤40 kPa.	W1N1Y1	**W1N1Y2**	**W1N2Y1**	W1N2Y2
	W2: 3 irrigations from emergence to panicle initiation, and one additional irrigation between panicle initiation and flowering.	**W2N1Y1**	W2N1Y2	W2N2Y1	**W2N2Y2**

**Table 5:** The variety names and codes used in the simulations. V1 to V7 denote varieties while S1 and S2 denote the seasons (i.e., 2011 and 2012, respectively). I and D indicate two growth environments: fully irrigated and rainfed at reproductive stage, respectively. The codes in bold indicate the datasets used for calibration.

Variety	Season 2011		Season 2012		Suitable environment^a^
	Irrigated	Drought	Irrigated	Drought	
FFZ	V1S1I	V1S1D	V1S2I	V1S2D	Irrigated
GSR IR1-12-DT10-SAL1-DT1	**V2S1I**	**V2S1D**	V2S2I	V2S2D	Drought and salt prone
GSR IR1-5-*S*AL10-DT1-DT1	**V3S1I**	**V3S1D**	V3S2I	V3S2D	Drought and salt prone
GSR IR1-8-*S*AL12-Y2-DT1	V4S1I	V4S1D	**V4S2I**	**V4S2D**	Drought and salt prone
IR74371-70-1-1	**V5S1I**	**V5S1D**	V5S2I	V5S2D	Drought prone irrigated
IR83142-B-19-B	V6S1I	V6S1D	**V6S2I**	**V6S2D**	Drought prone
PSBRc82	V7S1I	V7S1D	**V7S2I**	**V7S2D**	Irrigated

a The varieties suitable for the stressed environment are also good for the irrigated environment.

**Table 6:** The statistical results generated from the regression, Student’s *t*-test, error and modeling efficiency between simulated and measured values using ORYZA (v3) and ORYZA2000 (v2.13) for simulations.

Statisted variable^a^	N	Y	X	a	b	r^2^	P(t)	RMSEn	Meff
	ORYZA (v3)								
WAGT	341	4.75	4.75	0.32	0.95	0.98	0.92	12.34	0.98
WSO	161	2.89	2.88	0.20	0.91	0.98	0.43	23.23	0.96
WST	341	1.87	1.89	0.22	0.95	0.92	1.00	24.33	0.90
WLVD	175	0.80	0.86	0.14	0.69	0.85	1.00	48.06	0.78
WLVG	341	1.05	1.04	0.21	0.84	0.91	0.96	28.44	0.86
Yield	60	4.39	4.54	0.17	0.93	0.96	0.91	14.76	0.92
LAI	341	2.19	1.10	0.74	0.86	0.84	1.00	43.78	0.62
Leaf_N	168	1.09	1.10	0.39	0.63	0.72	0.38	29.57	0.50
AWD2	480	16.20	14.88	4.09	0.81	0.72	0.96	95.33	0.33
AWD3 + Aerobic3	1258	11.26	11.27	−0.01	1.00	1.00	1.00	1.06	1.00
AWD4	633	4.74	7.64	1.32	0.45	0.95	1.00	63.24	0.49
AWD5	639	6.87	6.88	0.00	1.00	1.00	0.33	0.93	1.00
AWD6	356	5.48	4.51	0.55	1.09	0.75	1.00	55.34	−0.12

	ORYZA2000 (v2.13)								
WAGT	341	4.99	4.75	0.37	0.96	0.97	0.99	22.51	0.95
WSO	161	2.65	2.88	0.42	0.89	0.96	0.95	28.19	0.91
WST	341	2.05	1.89	0.21	0.96	0.93	1.00	31.86	0.86
WLVD	175	0.71	0.86	0.15	0.73	0.85	0.98	55.19	0.72
WLVG	341	1.06	1.04	0.18	0.84	0.90	0.16	37.63	0.81
Yield	60	4.31	4.54	−0.19	0.99	0.93	0.94	20.48	0.84
LAI	341	2.46	2.05	0.69	0.79	0.84	1.00	48.64	0.69
Leaf_N	168	0.80	1.10	0.24	0.52	0.60	1.00	41.97	−0.12
AWD2	480	13.09	14.88	4.60	0.68	0.33	0.32	99.23	0.14
AWD3 + Aerobic3	1258	9.54	11.27	8.13	0.10	0.33	1.00	97.76	0.13
AWD4	633	12.91	7.64	3.61	1.04	0.28	0.99	400.20	−11.89
AWD5	639	7.56	6.88	5.38	0.32	0.31	0.99	93.02	−0.47
AWD6	356	9.92	4.51	5.27	0.67	0.55	1.00	74.87	−0.75

a The crop growth involved in the statistical analysis were WAGT: total above-ground biomass (t ha^−1^), WSO: panicle biomass (t ha^−1^), WST: stem biomass (t ha^−1^), WLVG: green leaf biomass (t ha^−1^), LAI: leaf area index, Leaf_N: nitrogen content of green leaves (g N m^−2^ leaf), and Yield: grain yield (t ha^−1^). The soil variables used for statistical analysis were AWD2, AWD4, AWD5 and AWD6: soil water potential in the 2nd, 4th, 5th and 6th soil layers AWD experiment, respectively, and AWD3 + Aerobic3: the water potential of the 3rd soil layer in AWD and ARE experiments.
