# Supplementary material for: From ORYZA2000 to ORYZA (v3): An improved simulation model for rice in drought and nitrogen-deficient environments
Source: Agric For Meteorol. 2017 May 1;237-238:246–56. doi: 10.1016/j.agrformet.2017.02.025 (PMC5391805; doi:10.1016/j.agrformet.2017.02.025)
Supplement: Supplementary file 1 [file mmc1.docx]

## Supplementary material

**From ORYZA2000 to ORYZA (v3): An improved simulation model for rice in drought and nitrogen-deficient environments**

Tao Li^*^, Olivyn Angeles, Manuel Marcaida III, Emmali Manalo, Mervin Pogs Manalili, Ando Radanielson, Samarendu Mohanty*

(International Rice Research Institute, Los Baños, Philippines)

## Model description and computation algorithms

### The computation of soil temperature dynamics

Soil temperature is an important factor that influences root growth and the processes of soil carbon and nitrogen. Thus, this module was developed to provide essential information for root and soil carbon and nitrogen modules.

The soil temperature module was developed based on the Fourier Law:

$\frac{{\partial T}_{i}}{\partial t}=D_{h,i}\frac{\partial^{2}T_{i}}{{\partial z}_{i}^{2}}=\frac{\lambda_{i}}{c_{h,i}}\frac{\partial^{2}T_{i}}{{\partial z}_{i}^{2}}$ (S1)

where *i* is the order of soil layer in the profile (Fig. 2a), *t* denotes time (seconds), *T_i_* is the soil temperature (^o^C), *D_h,i_* is the thermal diffusivity (m^2^ s^-1^), *λ_i_* is the thermal conductivity (J s^-1^ m^-1^ k^-1^), *c_h,i_* is the volumetric specific heat of the soil (J m^-3^ k^-1^), and *z_i_* is the depth (m) of the i^th^ soil layer.

Heat can be transferred between soil layers and can be stored in or taken from the storage of a soil layer (Fig. 2). For a given soil layer *i*, the equation S1 can be written as S2 (Campbell, 1985),

$K_{i}\left( \overline{T}_{i+1}-\overline{T}_{i} \right)-K_{i-1}\left( \overline{T}_{i}-\overline{T}_{i-1} \right)=c_{h}(T_{i}^{j+1}-T_{i}^{j})(z_{i+1}-z_{i-1})/(2\times\Delta t)$ (S2)

where *Δt* is the time increment, *j* is the superscript indicating the time at which temperature is determined, and
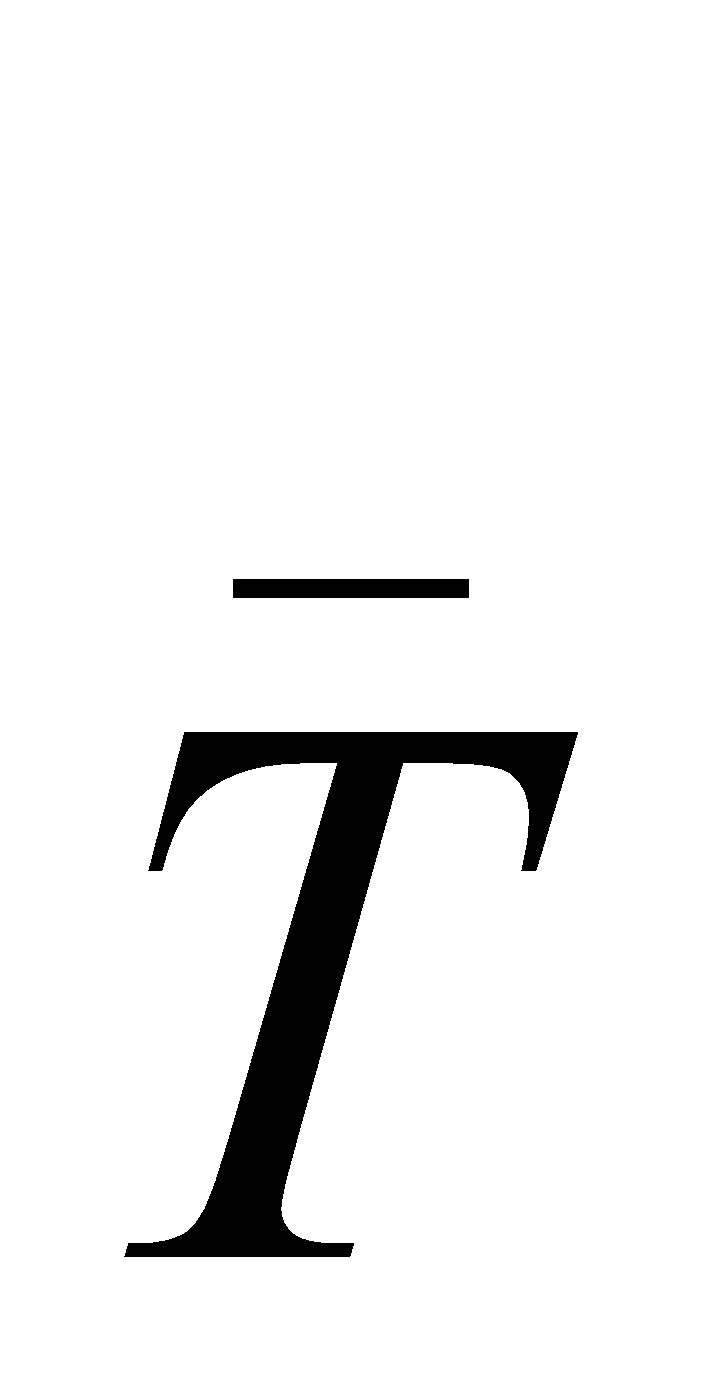
is the appropriate mean temperature (^o^C). Thermal conductance (*K_i_*, J s^-1^ m^-2^ k^-1^) is calculated from the thermal conductivity and the thickness of soil layer *i*.

$K_{i}=\frac{\lambda_{i}}{z_{i+1}-z_{i}}$ (S3)

In equation S2, if *T^j^* is the temperature at present time and *T^j+1^* is the temperature at the next time step, then
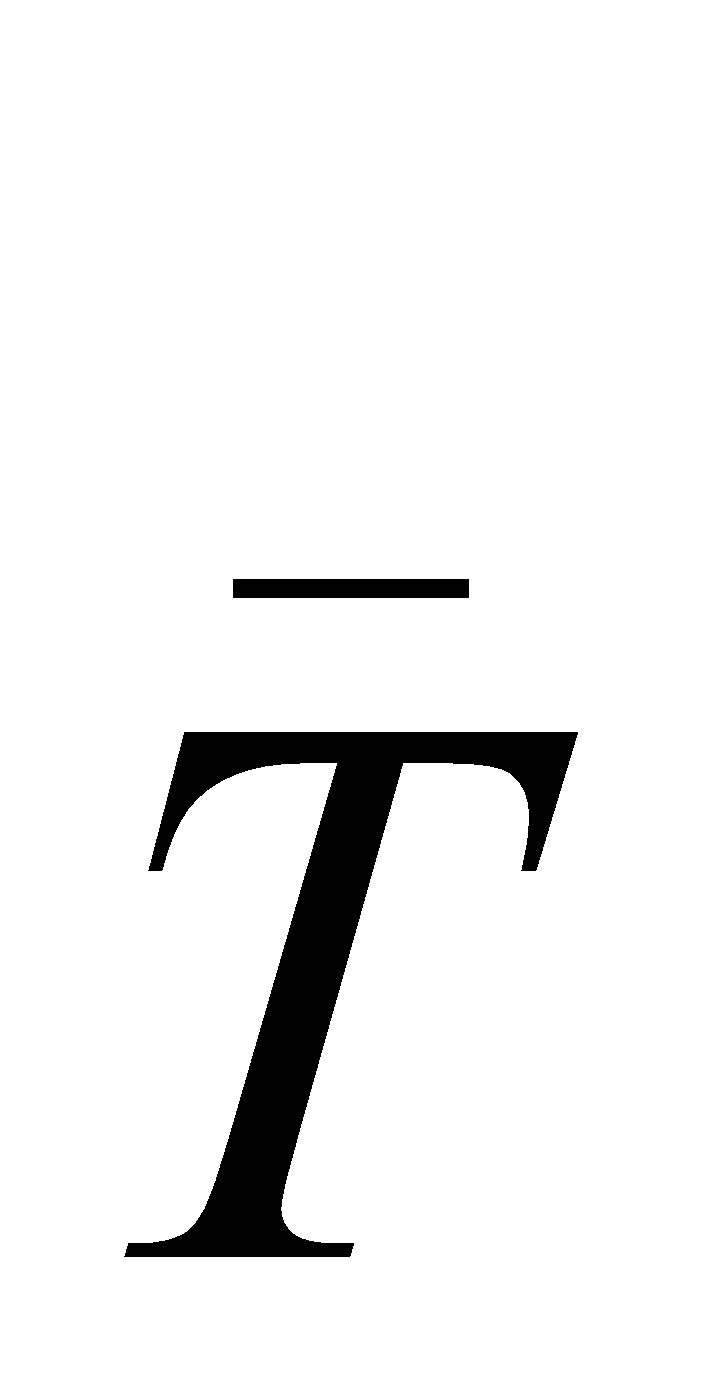
should be calculated from the temperature between *T^j^* and *T^j+1^*,

$\overline{T}={\eta T}^{j+1}+(1-\eta)T^{j}$ (S4)

where *η* is a weighing factor that ranges from 0 to 1.0.

From equation S2, stable numerical solutions are obtained only when $\Delta t<\frac{c_{h,i}{({\Delta z}_{i})}^{2}}{{2\lambda}_{i}}$ and *η* must be larger than 0.5 (Simonson, 1975).

Combining equations S2 and S4 for each soil node, the set of equations describing the system can be written in the following matrix format:

$\left[ \begin{aligned} \begin{matrix} B_{1} & C_{1} \\ A_{2} & B_{2} \end{matrix} \begin{matrix} 0 & 0 \\ C_{2} & 0 \end{matrix} \\ \begin{matrix} 0 & A_{3} \\ 0 & 0 \end{matrix} \begin{matrix} B_{3} & C_{3} \\ A_{4} & B_{4} \end{matrix} \end{aligned} \right]\left[ \begin{aligned} \begin{matrix} {TN}_{1} \\ {TN}_{2} \end{matrix} \\ {TN}_{3} \\ {TN}_{4} \end{aligned} \right]=\left[ \begin{aligned} \begin{matrix} D_{1} \\ D_{2} \end{matrix} \\ D_{3} \\ D_{4} \end{aligned} \right]$ (S5)

where

$A_{i}=C_{i}={-\eta K}_{i}$ (S6)

$B_{i}=\eta\left( K_{i}+K_{i-1} \right)+\frac{c_{h,i}(z_{i+1}-z_{i-1})}{2\Delta t}$ (S7)

and

$D_{i}=\left( 1-\eta\right)K_{i-1}T_{i-1}+\left( \frac{c_{h,i}(z_{i+1}-z_{i-1})}{2\Delta t}-\left( 1-\eta\right)\left( K_{i}+K_{i+1} \right) \right)T_{i}+(1-\eta)K_{i}T_{i+1}$ (S8)

For soil layer *i*, the new temperature at *j+1* is *TN_i_* and the old temperature at *j* is *T_i_*, while *D_i_* is the boundary temperature (^o^C). For the upper boundary, *D_1_* (which can be the water layer, residue layer, mixture layer of water and residue, or soil surface) is recalculated as

$D_{1}=D_{1}^{'}+{\eta K}_{0}T_{a}-R_{n}+LE$ (S9)

where$D_{1}^{'}$ is the upper boundary temperature at the previous time step (^o^C), *T_a_* is the present air temperature (^o^C), *R_n_* is the net radiation (J s^-1^ m^-2^) reaching the system surface, and *LE* is the latent heat flux (J s^-1^ m^-2^) (Campbell, 1985).

The lower boundary is assumed to be at constant temperature with zero heat flux, at which the *T_i+1_* in equation S8 is set as the average annual temperature of the soil.

Thermal conductivity *λ_i_* and *c_h,i_* are the unknown parameters to solve equations S1 to S9 (Campbell, 1985). They are not available from the measured observations but can be estimated from soil physical properties by numerical functions as follows (McInnes, [1981)](#_3o7alnk):

$\lambda_{i}={a+b\theta}_{i}-\left( a-d \right)\exp^{(-({{c\theta}_{i})}^{4})}$ (S10)

$a=0.65-0.78B_{i}+0.60B_{i}^{2}$ (S11)

$b=1.06B_{i}\theta_{i}$ (S12)

$c=1.0+2.6{(C_{i}^{clay})}^{-0.5}$ (S13)

$d=0.03+0.1B_{i}^{2}$ (S14)

where *B_i_* is the soil bulk density (Mg m^-3^) and *C_i_^clay^* is the fraction of clay content (g g^-1^).

Volumetric specific heat (*c_h,i_*) can be calculated from soil physical components,

$c_{h,i}=\left( 2.39\left( 1-\frac{B_{i}}{2.65} \right)+4.18\theta_{i} \right)*{10}^{6}$ (S15)

### Carbon and nitrogen dynamic module

#### *Carbon decomposition and nitrogen mineralization*

The decomposition of fresh organic carbon is calculated by a zero-order function for each soil layer in the module,

$C_{t}=C_{t-1}(1-K_{t}\delta_{t})$ (S16)

where *C_t_* is the fresh organic carbon content (g C m^-3^) at time t, *C_t−1_* is the fresh organic carbon content in an earlier time step, *K_t_* is the actual decomposition rate (d^-1^), and *δ_t_* is the time step.

The actual decomposition rate (*K_t_*) is modified from the potential decomposition rate (*k*) by soil water content and temperature conditions as well as by the quality of fresh organic matter,

$K_{t}=k\times f\left( T_{s} \right)f(\theta)f(R_{CN})$ (S17)

where *f(T_s_)* is a function of soil temperature (*Ts*, ^o^C), *f(θ)* is a function of soil water content (*θ*, cm^3^ cm^-3^), and *f(R_CN_)* is a function of the organic matter C to N ratio (*R_CN_*) used to modify carbon decomposition and mineralization (Eqs. S18 to S20) ([Godwin and Jones, 1991;](#_2xcytpi) Kirschbaum, 1995; [Vigil and Kissel, 1991](#_3fwokq0)[)](#_qsh70q).

This module assumes that *k*, the potential decompose rate (d^-1^), is a constant similar to CERES ([Godwin and Jones, 1991)](#_2xcytpi), in which freshly incorporated organic matter contains 20% carbohydrates, 70% cellulose, and 10% lignin, with maximum decay rates of 0.2, 0.05, and 0.0095, respectively. In this module, the composition of rice straw is 54% carbohydrates, 38% cellulose, and 8% lignin, while the composition of root is 65% carbohydrates, 28% cellulose, and 7% lignin ([Wu et al., 2009)](#_2u6wntf), which has the same default decomposition rates as those used in CERES. *θ_FC_*, *θ_r_*, and *θ_s_* are the soil water content at field capacity, the residual soil water content, and the saturated soil water content (cm^3^/cm^3^), respectively. *R_CN_* is the C:N ratio of fresh organic matter and *R_CNmax_* is the maximum C:N ratio of stabilized humus. The default C:N ratio for straw is 39:1 (38% C and 0.97% N) whereas, for root, the default C:N ratio is 23:1 (31.2% C and 1.33% N) in this module ([Wu et al., 2009)](#_2u6wntf):

$f\left( T_{s} \right)=0.010645\times e^{(0.12979\times T_{s})}$ (S18)

$f\left( \theta\right)=\left\{ \begin{aligned} 1.0-\frac{\theta-\theta_{FC}}{\theta_{s}-\theta_{FC}} \theta\geq\theta_{FC} \\ \frac{\theta-\theta_{r}}{\theta_{FC}-\theta_{r}} &\theta<\theta_{FC} \end{aligned} \right.$ (S19)

$f\left( R_{CN} \right)=e^{-0.693\frac{R_{CN}-R_{CN max}}{R_{CN max}}}$ (S20)

A part of the decomposed fresh organic carbon will be released back into the atmosphere in the form of carbon dioxide (CO_2_) and the other part will be converted into humus-carbon for the establishment of a soil organic carbon stock. In this module, 60% of the decomposed carbon will be CO_2_ emissions and 40% will be used by microbes for carbon fixation (Vigil et al., 1991).

Associated with the decomposition of organic carbon and organic nitrogen is mineralization. The module presumes that the mineralization rate of organic matter also follows the same first-order function of carbon decomposition (Eq. S16). In equation S16, the organic carbon content is replaced by organic nitrogen content, and the mineralization rate is the same as the decay rate. However, if the organic nitrogen in the fresh organic matter is not enough to maintain the potential carbon decomposition, the immobilization of mineral nitrogen will occur which is computed as follows ([Feng and Li, 2001)](#_1y810tw):

$N_{im}=min(\max(0.0,\delta_{c}\left( {R^{'}}_{n:c}-\frac{N_{t-1}}{C_{t-1}} \right),(N_{NH4}+N_{NO3}-N_{NH4}^{'}-N_{NO3}^{'}))$ (S21)

where *δ_c_* is the total carbon decomposed (g C m^-3^),
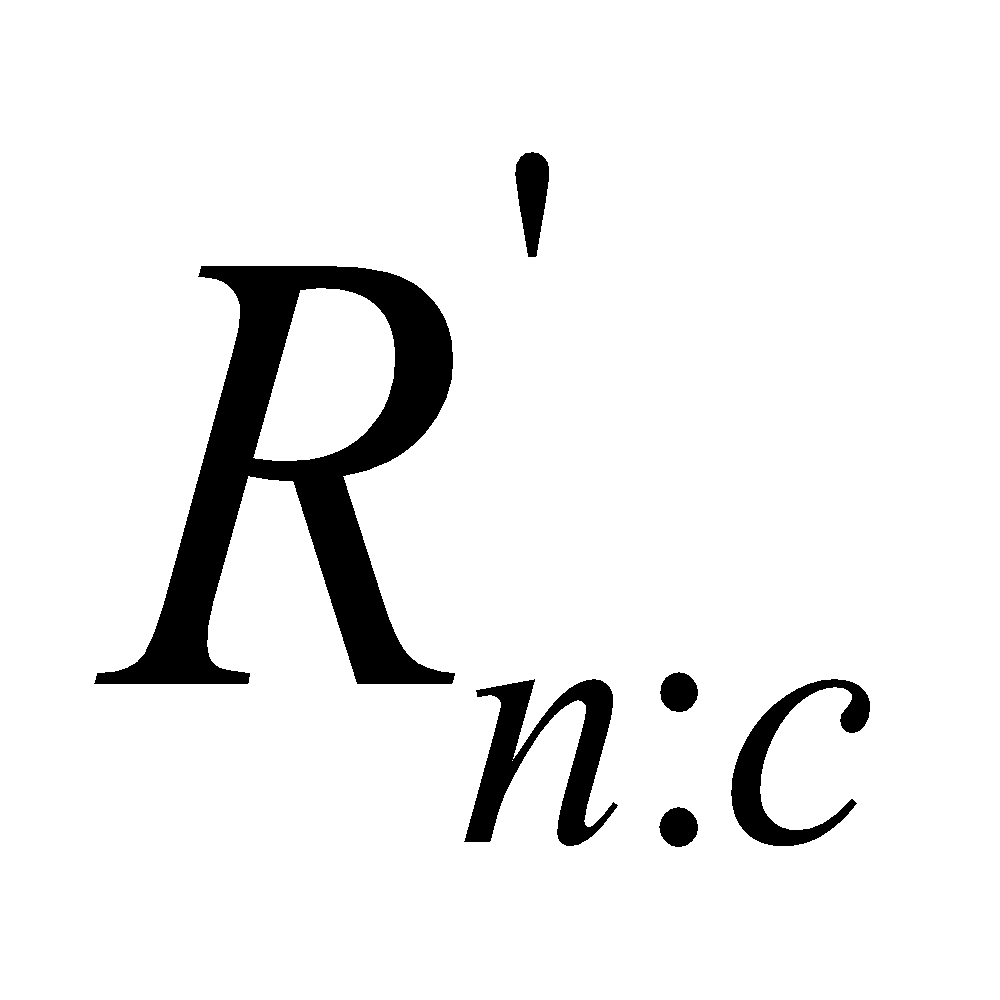
is the N:C ratio of stable soil organic matter (1:11 normally), *C_t−1_* is the carbon content in fresh organic matter at a previous time (g C m^-3^), *N_t – 1_* is the nitrogen content in fresh organic matter at a previous time (kg N m^-3^), *N_NH4_* and *N_NO3_* are the ammonium- and nitrate-nitrogen contents in soil, respectively, and *N^’^_NH4_* and *N^’^_NO3_* are the reserve amounts of ammonium- and nitrate-nitrogen that the plant and microbes could not uptake (g N m^-3^).

The third term in equation S21 determines the maximum amount of mineral nitrogen available for immobilization in the soil. The second term determines whether organic nitrogen is enough for carbon decomposition (*δ_c_*).

The net mineralized nitrogen is the difference between the mineralized and immobilized nitrogen:

$N_{net}=max(0.0,\delta_{c}\frac{N_{t-1}}{C_{t-1}}-N_{im})$ (S22)

The organic nitrogen is updated in equation S23:

$N_{t}=N_{t-1}+(N_{im}-\delta_{c}\frac{N_{t-1}}{C_{t-1}})$ (S23)

At the same time, soil mineral nitrogen content is recalculated as follows:

$N_{NH4,t}=N_{NH4,t-1}+N_{net}-N_{im}\frac{N_{NH4,t-1}-N_{NH4}^{'}}{N_{NH4,t-1}+N_{NO3,t-1}-N_{NH4}^{'}-N_{NO3}^{'}}$ (S24)

$N_{NO3,t}=N_{NO3,t-1}-N_{im}\frac{N_{NO3,t-1}-N_{NO3}^{'}}{N_{NH4,t-1}+N_{NO3,t-1}-N_{NH4}^{'}-N_{NO3}^{'}}$ (S25)

where *N_NH4,t_* and *N_NO3,t_* are the ammonium and nitrate contents in current time steps (kg N m^-3^); *N_NH4,t-1_* and *N_NO3,t−1_* are the ammonium and nitrate contents in previous time steps (kg N m^-3^); and *N_net_* is the mineralized nitrogen in current time step (kg N m^-3^).

Similar computation algorithms apply for carbon decomposition and mineralization in humus. The decomposition of humus-carbon is slower than fresh organic carbon. Equations S16 to S20 are also used to calculate the decomposition of humus by using a lower potential decomposition rate (i.e., 0.000083 d^-1^) ([Godwin and Jones, 1991)](#_2xcytpi). This module assumes that immobilization does not exist for humus, that is, *N_im_* is zero for humus. Equations S21 to S25 are also used to calculate mineralized nitrogen, humus-nitrogen content, and soil mineral-nitrogen content with *N_im_ = 0.0*. Therefore, the total carbon loss is the sum of carbon lost from the decomposition of fresh organic carbon and humus while total soil organic carbon is the sum of the remaining fresh organic carbon and humus.

#### *Urea hydrolysis*

The process of hydrolysis starts after the application of urea fertilizer. This module assumes that surface fertilizer, urea, is uniformly incorporated into the topsoil layer.

The actual hydrolyzed amount (*g N m^-2^*) in each soil layer is estimated by equation S26 and the product as NH_4_^+^ is added into the soil in the corresponding soil layer (Eq. S27). The hydrolyzed amount is deducted from urea (Eq. D28),

$H_{[Urea]}=min(\left[ Urea \right],f_{\left[ Urea \right]}f_{w}f_{T}[Urea])$ (S26)

$\left[ {NH}_{4} \right]=\left[ {NH}_{4} \right]+H_{[Urea]}$ (S27)

$\left[ Urea \right]=\left[ Urea \right]-H_{[Urea]}$ (S28)

where *H_[Urea]_* is the hydrolyzed amount (g N m^-2^), *[Urea]* is the content of urea (g N m^-2^), *[NH_4_]* is the content of NH_4_ (g N m^-2^), *f_[Urea]_* is the potential hydrolyzing rate (g N m^-2^), *f_w_* is the modified function of soil water to hydrolysis*,* and *f_T_* is the modified function of soil temperature to hydrolysis in a soil layer.

The potential hydrolysis rate, *f_[Urea]_*, is estimated from the soil organic carbon (*TOC*) and the pH (*pH*) of a soil layer (Eq. S29) using a modified function used in CERES ([Godwin and Jones, 1991)](#_2xcytpi):

$f_{[Urea]}=max(h_{min},(-1.12+0.203pH+(0.524-0.062pH)TOC))$ (S29)

The maximum hydrolysis rate in a soil layer is scaled down according to the water conditions (Eq. S30) and soil temperature of this soil layer (Eq. S31) ([Godwin and Jones, 1991)](#_2xcytpi),

$f_{w}=\left\{ \begin{aligned} \frac{\theta-\theta_{r}}{\theta_{FC}-\theta_{r}} \theta\leq\theta_{FC} \\ \frac{\theta_{s}-\theta}{\theta_{s}-\theta_{FC}} \theta>\theta_{FC} \end{aligned} \right.$ (S30)

$f_{T}=min(1,\max\left( 0,\frac{T^{s}}{h_{2}^{T}}+h_{1}^{T} \right))$ (S31)

The *h_min_* is the minimum hydrolyzing rate for active hydrolysis, *h^T^_1_* is the compensation temperature coefficient for hydrolysis at low temperature, and *h^T^_2_* is the maximum soil temperature for active hydrolysis (^o^C). The maximum hydrolysis rate is normally set as 0.25 ([Godwin and Jones, 1991)](#_2xcytpi). The *f_T_* will have value for 1.0 if the *T^s^*  is higher than *h^T^_2_*.

#### *Nitrification*

The nitrification is an aerobic process which relates to NH_4_ content (g N m^-2^), soil water, soil temperature, and depth of soil layer (Eq. S32).

$N_{[NH4]}=[{NH}_{4}](max(0,min(0.1,f_{T}f_{w}f_{d}f_{[nh4]})))$ (S32)

Nitrification rate will decrease exponentially with a decrease in ammonium content in the soil when the ammonium content is lower than 0.1 kg N m^-3^ soil. Otherwise, the ammonium content will not have an effect on the nitrification rate,

$f_{[{NH}_{4}]}=\left\{ \begin{aligned} 1.0 &[{NH}_{4}]\geq z \\ 1.0-\exp\left( -\frac{\left[ {NH}_{4} \right]}{0.1z} \right) [{NH}_{4}]<z \end{aligned} \right.$ (S33)

where *[NH_4_]* is the ammonium content (g N m^-2^) and *z* is the thickness (m) of a soil layer to account for the effect of ammonium concentration in a given soil volume on nitrification.

Nitrification is an aerobic process; therefore, the exchange of O_2_ in the soil affects the nitrification rate. Aeration decreases with an increase in the depth of the soil layers, leading to a lower supply of oxygen. In this module, nitrification is assumed to be reduced by 55% with an increment of 30-cm soil depth,

$f_{d}={(0.45)}^{\frac{d}{0.3}}$ (S34)

where *d* is the depth (m) of a soil layer and *f_d_* is the factor of soil depth to scale down the nitrification rate.

Nitrification is a biological process that is strongly affected by soil temperature and water content. The function for soil temperature from Vigil and Kissel (1995) is used to modify the nitrification rate:

$f_{T}=1.0-exp(-\exp\left( 24.635-\frac{8227.000}{T^{s}+237.160} \right))$ (S35)

where *T^s^* is the soil temperature (^o^C) to scale down the nitrification rate in a soil layer and *f_T_* is the factor of soil temperature to scale down the nitrification rate in the soil layer*.*

The soil water content function used in the nitrification process is the same as the function used in urea hydrolysis. Nitrates are not the only products of nitrification. Nitrous oxide (N_2_O) is also a product of nitrification although its amount is only 2% of the total nitrified nitrogen. Therefore, the daily ammonium and nitrate content as well as the amount of N_2_O in a soil layer are updated daily for each soil layer,

$[{NH}_{4}]=[{NH}_{4}]-N_{[NH4]}$ (S36)

$\left[ {NO}_{3} \right]=[{NO}_{3}]+{0.98N}_{[NH4]}$ (S37)

$\left[ N_{2}O \right]={0.02N}_{[NH4]}$ (S38)

#### *Denitrification*

Denitrification is an anaerobic process and this module assumes that denitrification will occur only if the soil water content is higher than the field capacity. Taking into account the effects of soil water content and temperature on denitrification, the actual denitrification in the soil layer *i* (*D_Ni_*, g N m^-2^) is calculated as,

$D_{N}={\gamma C}_{w}[{NO}_{3}]f_{w}f_{T}$ (S39)

where *C_w_* is the corresponding coefficient between soluble carbon and denitrification in a soil layer (g C m^-2^); and $\gamma$ is the maximum proportion of nitrate can be denitrified at optimal water and temperature with given soluble carbon ((g C m^-2^)^-1^).

Denitrification provides an electron acceptor for soil carbon decomposition. Indeed, it is coupled with the decomposition of carbon. However, this module also assumes that denitrification will not occur if there is no soluble carbon in the soil layer. Soluble carbon content in a soil layer is estimated following Rolston et al. (1980) from total soil organic carbon content(*TOC*, g C m^-2^),

$C_{w}=\left\{ \begin{aligned} 0 &TOC=0 \\ 24.5+3.1\frac{TOC}{10z} &TOC>0 \end{aligned} \right.$ (S40)

The denitrification rate increases linearly with an increase in soil water content after soil water content exceeds the field capacity (Eq. S41). Soil temperature is exponentially related to the denitrification rate when the temperature is lower than 50^o^C (Eq. S42) (Godwin and Jones, 1991).

$f_{w}=max(0,\frac{\theta-\theta^{fc}}{\theta^{s}-\theta^{fc}})$ (S41)

$f_{T}=min(1,0.1exp(0.046T^{s}))$ (S42)

The concentration of nitrate in a soil layer is updated after each time step of the denitrification process (S43).

$\left[ {NO}_{3} \right]=\left[ {NO}_{3} \right]-D_{N}$ (S43)

The products of denitrification include a mixture of NO_2_^–^, NO^−^, N_2_O, and N_2._ NO_2_^−^ and NO^−^ are intermediate and unstable products. N_2_O is the first end product. N_2_ will be produced when the requirement for an electron acceptor in the oxidation process cannot be achieved by the reduction process from NO_3_^−^ to N_2_O. In most cases, the nitrogen gases generated from denitrification contain a mixture of N_2_O and N_2_. Carbon decomposition (carbon oxidation), the source of denitrification (i.e. NO_3_^−^ content), and soil water content affect the ratio of N_2_O and N_2_ in the nitrogen gas emissions,

$R_{N2/N2O}=\min\left( f_{r}\left( {NO}_{3} \right),f_{r}\left( {CO}_{2} \right) \right)f_{r}(\theta)$ (S44)

where *R_N2/N2O_* is the ratio of N_2_ to N_2_O in nitrogen gas, *f_r_(NO_3_)* is the function to determine the ratio of N_2_ to N_2_O by nitrate content (kg N m^-2^) (Eq. S45), *f_r_(CO_2_)* is the function to determine the ratio of decomposed carbon (g C m^-2^) (Eq. S46), and *f_r_(θ)* is the function to determine the ratio of soil water content in a soil layer (Eq. S47) (Del Grosso et al., 2000).

The amount of N_2_O (*N_N2O_*, g N m^-2^) generated from the denitrification process is calculated from the denitrified amount and the N_2_ to N_2_O ratio (Eq. S48) (Del Grosso et al., 2000),

$f_{r}\left( {NO}_{3} \right)=max(0.0,25-(0.5+\frac{a\tan(\pi\times0.01\times([{NO}_{3}]\times10000-190))}{\pi}))$ (S45)

$f_{r}\left( {CO}_{2} \right)=max(0.0,13+\frac{30.78\times a\tan(\pi\times0.07\times(\delta_{c}\times10000-13))}{\pi})$ (S46)

$f_{r}\left( \theta\right)=\frac{1.4}{(\frac{17}{{13}^{{13}^{(2.2\times\theta/\theta^{s})}}})}$ (S47)

$N_{N2O}=D_{N}\times\frac{1}{1+R_{N2/N2O}}$ (S48)

### Root growth

The living root carbon (*R_c_*, kg C ha^-1^) in a soil layer is the difference between the accumulated root carbon allocated (*A_c_*, kg C ha^-1^) and the total senescence (D_c_, kg C ha^-1^) in the soil layer,

$R_{c}=A_{c}-D_{c}$ (S49)

and *A_c_*  is the daily accumulation of new root carbon,

$A_{c}=A_{c}^{'}+\delta_{c}$ (S50)

where$A_{c}^{'}$ is the allocated root carbon on the previous day (kg C ha^-1^) and *δ_c_* is the new root carbon (kg C ha^-1^) in a soil layer stratified from the total new root carbon on a given day.

The new assimilate allocated into the roots is stratified into the soil layers according to the distribution factor. The *δ_c,i_* is the specified *δ_c_* for the soil layer *i*, which is calculated as

$\delta_{c,i}=R_{c}\frac{f_{i}}{\sum_{i=1}^{n} f_{i}}$ (S51)

where *R_c_* is the total carbon allocated into the root from assimilate (kg C ha^-1^), *f_i_* is the distribution factor (unit-less) in the soil layer *i*, and *n* is the number of rooted soil layers.

The routine in the model assumes that most roots are in the upper soil layers and root biomass exponentially decreases with soil depth (Schenk, 2008; [Wulfsohn et al., 1996](#_19c6y18)[)](#_1hmsyys). Soil properties and environmental conditions affect root growth and root distribution within the soil profile. The distribution factor for the i^th^ soil layer (*f_i_*) is calculated from an exponential function and modified by factors of soil environment in this soil layer (Eq. S52). This equation is adopted from Li at al. (2009) and modified for rice.

$\left\{ \begin{aligned} f_{i}^{'}=\sum_{x=ul}^{ll} (\exp\left( -kd_{x.i} \right)\left( t_{i}+a_{i}+p_{i}+w_{i}+n_{i} \right)) \\ f_{i}=f_{i}^{'}/\sum_{i=1}^{rl} f_{i}^{'} \end{aligned} \right.$ (S52)

Where $f_{i}^{'}$ is the numeric number of the i^th^ soil layer for computing the relative new root distribution in rooting profile, *rl* is the number of soil layers of rooting profile in a given time, *d_x,i_* is the depth of calculation from the soil surface, *x* represents changes from the upper (*ul*) to the lower (*ll*) boundary of soil layer *i*, *k* is the extinction coefficient of the new root distribution in the soil profile, and *p_i_*, *t_i_*, *w_i_,* and *n_i_* are the soil penetration, temperature, water, and nitrogen factors, respectively, that influence root distribution.

The routine presumes that 95% of the new carbon would be allocated within the soil profile constrained by the potential root depth. Therefore, parameter *k* in equation S54 can be calculated as

$k=-ln(0.05)/(max(d_{max},d_{max}DVS,d^{'}))$ (S53)

where *DVS* is the development stage ranging from 0 to 2, *d^’^* is transplanting or direct-seeding depth (cm), and *d_max_* is a genetic parameter representing the maximum rooting depth of a variety (cm).

The routine assumes that rice reaches its maximum rooting depth during its vegetative stage (i.e., DVS ≤1.0) because little assimilate can be allocated into the root during the reproductive stage to maintain the root system built up at the vegetative stage. The model assumes that the downward development of the root is constant under optimal conditions. The *d_max_ DVS* indicates the moment potential depth (cm) of the rooting frontier in the vegetative stage, which increases parallel to plant development.

Root growth and distribution can be limited by soil properties and environmental conditions. Soil texture, aggregation, and organic matter content change slowly. Soil temperature, soil water content, mineral nitrogen concentration, and air condition are dynamic, changing significantly from day to day. These factors adjust the distribution of roots in the soil profile by changing the allocation of new root carbon.

Roots grow very slowly under a low soil temperature condition ([Chaudhary et al., 1985;](#_z337ya) [Jakobsen and Dexter, 1987)](#_3whwml4). Jones et al. ([1991)](#_2bn6wsx) used a sine function to account for the soil temperature suitability on root growth in the i^th^ soil layer,

$t_{i}=\frac{T_{i}}{T_{max}}$ (S54)

$T_{i}=\left\{ \begin{aligned} \sin(1.57\frac{T_{i}^{s}-T_{min}^{s}}{T_{opt}^{s}-T_{b}^{s}}) &T_{i}^{s}\geq T_{min}^{s} \\ 0 &T_{i}^{s}<T_{min}^{s} \end{aligned} \right.$ (S55)

where *t_i_* is the temperature suitability factor ranging from 0 to 1.0 and the layer with the most suitable temperature for root growth is 1.0. *T_i_* and $T_{i}^{s}$ (^o^C) are the index of the most suitable soil temperature for root growth and soil temperature in the i^th^ soil layer, respectively. For root growth, which is a genotype-dependent parameter, $T_{min}^{s}$ (^o^C) is the lowest soil temperature for root survival, $T_{opt}^{s}$ (^o^C) is the optimum soil temperature, $T_{b}^{s}$is the lowest soil temperature, and *T_max_* is the maximum soil temperature. Equation S55 was developed from the results generated by Stone and Taylor ([1983)](#_41mghml) and Voorhees et al. (1981).

Root growth is sensitive to soil air conditions. Plants have different behaviors regarding their adaptation to unfavorable air conditions, especially at low oxygen concentration in their rhizosphere. A low oxygen exchange rate can significantly reduce root density in the soil ([Watson and Kelsey, 2006)](#_1v1yuxt). The development of suboptimal oxygen concentrations is affected by several soil characteristics such as texture, porosity, water content, biotic activity, temperature, surface-water movement, continuity of air-filled pores, land management, and soil compaction ([Benjamin et al., 2003;](#_lnxbz9) [Drew, 1983;](#_3j2qqm3) [Grable, 1966;](#_1ci93xb) [Micucci and Taboada, 2006;](#_23ckvvd) [Watson and Kelsey, 2006)](#_1v1yuxt). The simulating algorithm of the air effects on root growth was adopted from the functions developed by Jones et al. (1991). The air factor for rice root growth in i^th^ soil layer (*a_i_*) is a relative value among rooted soil layers and is factored to be 0 to 1.0,

$a_{i}=\frac{A_{i}}{A_{max}}$ (S56)

where *A_i_* is the air effect on the root growth in soil layer *i* and *A_max_* is the maximum value of all *A_i_* within the rooted soil layers. The *A_i_* is calculated from the ability to tolerate saturated conditions (*F^’^*), saturated degree (*S_i_^w^*), and the critical water-filled porosity of soil layer *i* (*F_i_^w^*) (Jones et al., 1991; Li et al., 2009).

$A_{i}=\left\{ \begin{aligned} F^{'}+\frac{(1-S_{ij}^{w})(1-F^{'})}{1-F_{i}^{w}} &S_{i}^{w}>F_{i}^{w} \\ 1.0 &S_{i}^{w}\leq F_{i}^{w} \end{aligned} \right.$ (S57)

There is no reduction in root growth in saturated soil conditions if *F^’^* is 1.0. Rice is a typical example of tolerance in low-oxygen conditions. There is no root growth in saturated soil if *F^’^* is 0. The value of *F^’^* cannot be set to 0 for rice because it is not a flood-sensitive plant like maize, cotton, soybean, wheat, and sunflower. The term *F_i_^w^* is determined by the clay content of soil layer *i* ($C_{i}^{clay}$),

$F_{i}^{w}=0.4+0.004C_{i}^{clay}$ (S58)

Air-filled porosity (*1.0 – S_i_^w^*) is related to the soil air condition and it affects root growth by significantly changing O_2_ diffusivity, O_2_ concentration, and soil redox potential (Benjamin et al., [2003;](#_lnxbz9) Watson and Kelsey, [2006)](#_1v1yuxt). The *S_i_^w^* is the ratio of the volumetric soil water content to the total porosity of soil layer *i* (*θ_i_* and *θ_i_^s^*):

$S_{i}^{w}=\frac{\theta_{i}}{\theta_{i}^{s}}$ (S59)

Soil penetrability, which is the condition for root penetration in a soil profile, has an approximately linear relationship with root growth (Busscher and Bauer, 2003; [Gerard et al., 1982;](#_4i7ojhp) [Laboski et al., 1998;](#_3as4poj) [Vepraskas and Miner, 1986;](#_vx1227) [Zou et al., 2001](#_28h4qwu) [)](#_44sinio). It is mainly determined by soil properties such as bulk density, texture, soil water content, and land management for tillage and compaction ([Jakobsen and Dexter, 1987;](#_3whwml4) [Lampurlanés et al., 2001;](#_1pxezwc) Lipiec et al., 2003; Lipiec and Hatano, 2003; [Salih et al., 1998](#_32hioqz)[)](#_147n2zr). Bulk density, texture, and soil water content are considered in the estimation of soil penetrability in the root growth computation. Parameter *p_i_* in equation S52 represents the factor of soil penetrability, which is the fraction of penetrability of soil layer *i* to the maximum value of penetrability of the rooted profile,

$P_{i}=\frac{{SP}_{i}}{{SP}_{max}}$ (S60)

where *SP_i_* is the penetrability of the root in soil layer *i* (MPa^-1^) and *SP_max_* is the maximum of all *SP_i_* within rooted soil layers.

Soil penetrability is calculated from soil moisture, clay content, and bulk density (Eqs. S61 to S65) ([Canarache, 1990)](#_2jxsxqh),

${SP}_{i}=\frac{1}{P_{i}^{'}}{(\frac{S_{i}\rho_{i}}{{2\theta}_{i}})}^{m_{i}}$ (S61)

$m_{i}=0.36\times{1.0026}^{C_{i}^{clay}}\times B_{i}^{1.27}\times B_{i}^{{0.267C}_{i}^{clay}}$ (S62)

$\rho_{i}=1.195+12.0755\frac{1-B_{i}}{44.9+{0.163C}_{i}^{clay}}$ (S63)

$S_{i}=\frac{1-{0.38B}_{i}}{B_{i}}\times100.0$ (S64)

$P_{i}^{'}=0.055\times{1.047}^{C_{i}^{clay}}B_{i}^{7.53}$ (S65)

where *m_i_*, *S_i_*, and *ρ_i_* are intermediate variables, *P_i_^’^* represents the initial soil penetration resistance (MPa), *C_i_^clay^* is the clay content (%), *B_i_* is bulk density (g cm^-3^), and *θ_i_* is the volumetric moisture content (%) of soil layer *i*.

The plant needs water and nitrogen for growth and these are extracted from the soil through the root. Water and nitrogen also influence root growth and root distribution in the soil profile. Generally, the root grows toward the place where water and nitrogen are relatively rich. The rice root growth sub-model assumes that the favorable moisture condition for root growth is saturation and no growth occurs when the soil water content is lower than the wilting point. Root growth decreases linearly with the decrease in soil water content. The term *w_i_* in equation S52 indicates the relatively favorable moisture condition for root growth:

$W_{i}=\frac{W_{i}}{W_{max}}$ (S66)

where *w_i_* is the moisture effect on root growth in soil layer *i* and *W_max_* represents the maximum *W_i_* among all values within root soil layers. *W_i_* of soil layer *i* is calculated from the soil moisture content, saturated water content, and wilting point of soil layer *i* (*θ_i_*, *θ_j_^s^* and *θ_j_^wp^*).

$W_{i}=\left\{ \begin{aligned} 0 &\theta_{i}\leq\theta_{i}^{wp} \\ \frac{\theta_{i}-\theta_{i}^{wp}}{\theta_{i}^{s}-\theta_{i}^{wp}} &\theta_{i}^{wp}<\theta_{i}<\theta_{i}^{s} \end{aligned} \right.$ (S67)

The term *n_i_* in equation S52 represents the relative richness of nitrogen,

$n_{i}=\frac{N_{i}}{N_{max}}$ (S68)

where *N_i_* is the mineral nitrogen content (kg N ha^-2^) in soil layer *i* and *N_max_* is the maximum *N_i_* (kg N ha^-1^) within the rooted soil layers.

### Water and nitrogen uptake and stress quantification

#### *Water uptake*

To estimate the water uptake, the module assumes that (1) the root can absorb all extractable soil water to meet the potential transpiration to achieve maximum assimilation; (2) the water uptake rate per unit of root mass is the same in all soil layers, differences of aeration and/or air content in the soil layers do not then affect water uptake; and (3) the drought tolerance factor (DTF) adjusts the extractability of soil water as well as assimilation rate. The DTF, a genetic parameter, is the inverse of the ratio of available soil water at which plant transpiration starts to decline to the maximum available soil water content (Fig. S1). Figure 1 illustrates the definition of DTF, which is cultivar-specific but environment-independent.

Figure S1. An illustration of the relationship between soil water content and transpiration.The data used in this figure were derived from the experiment with variety IR72 in the 1992 dry season (Wopereis et al., 1996). The soil water potential was converted into soil water content using the Van Genuchten function, and the parameters for the soil and the fraction of actual transpiration to the potential transpiration were reclassified to maximum for 1.0.

The maximum available soil water is the difference between the saturated (SWCST) and wilting point (SWCWP) soil water contents. DTF indicates the threshold at which transpiration will decline significantly if soil water content decreases continuously towards SWCWP.

In this module, the amount of extractable soil water decreases with the decrease in soil water content. The energy needed to break the adsorption of water by soil particles at lower soil water content is higher than in saturated soil. The physically available water (the water amount between saturated and wilting point) is partially extractable for the plant’s use ([Azhar and Perera, 2006)](#_26in1rg). Therefore, the rate of availability of the extractable water (*w_e,i_*) decreases with decreases in soil water content in soil layer *i*. It is determined by DTF (Fig. S2A):

$w_{e,i}=\left\{ \begin{aligned} \theta_{i}-\theta_{i}^{fc}+\int_{\theta_{i}^{wp}}^{\theta_{i}^{fc}} \left( 1.0-\left( \frac{\theta_{i}^{fc}-\theta_{x}}{\theta_{i}^{fc}-\theta_{i}^{wp}} \right)^{DTF} \right)\theta_{i}\geq\theta_{i}^{fc} \\ \int_{\theta_{i}^{wp}}^{\theta_{i}} \left( 1.0-\left( \frac{\theta_{i}^{fc}-\theta_{x}}{\theta_{i}^{fc}-\theta_{i}^{wp}} \right)^{DTF} \right)\theta_{i}<\theta_{i}^{fc} \end{aligned} \right.$ (S69)

where $\theta_{i}^{fc}$ is the soil water content at field capacity, $\theta_{i}^{wp}$ is the soil water content at wilting point, and *θ_i_* is the soil water content of i^th^ soil layer. The *θ_x_* is the variable in the integration function for soil water content varying between the integrating range.

**Figure S2.** Illustration of the relationship of (**A**) extractable soil water to the drought tolerance factor (DTF) and soil water content; and (**B**) the relationship of potential water uptake to the amount of extractable water and root mass.

The DTF and hydrologic parameters in Figure S2 are values to illustrate a graphical representation of the relationship in panel A: when soil water content is between field capacity and saturated condition, the physically available water above field capacity ($\boldsymbol{\theta}_{\boldsymbol{i}}\boldsymbol{-}\boldsymbol{\theta}_{\boldsymbol{i}}^{\boldsymbol{fc}}$ in equation S69) will be completely available for uptake. For panel **B**, the total root mass in the soil profile was assumed to be 600 kg/ha while the total water in the root zone was assumed to be 60 mm.

The potential uptake of the whole rooting profile (*U_w,p_*, mm) is calculated from the interaction of root biomass and the amount of extractable soil water,

$U_{w,p}=\sum_{i=1}^{n} W_{i}(\frac{W_{i}R_{c,i}}{\sum_{i=1}^{n} W_{i}R_{c,i}})$ (S70)

where *n* is the number of rooted soil layers and *R_c,i_* is the root biomass of soil layer *i* (kg C/ha).

However, the actual uptake of water (*U_w_*, mm) is the minimum value between water demand and potential uptake (Eq. S71). In the model, water demand is equal to potential transpiration (*T_p_*, mm).

$U_{w}=min(T_{p}, U_{w,p})$ (S71)

#### *Drought stress quantification*

Drought stress is quantified by an exponential function of the ratio of water uptake to demand (Eq. S72) ([Ronda et al., 2001; Li et al., 2009)](#_3znysh7). The drought stress index (*S_w_*) will be used to scale down potential photosynthesis into water-stressed photosynthesis (Fig. S3) and to adjust the assimilate allocation among plant organs,

$S_{w}=1.0-{(1.0-min (1.0-\frac{U_{w}}{T_{p}}) )}^{DTF}$ (S72)

DTF limits the rate of reduction in photosynthesis under drought stress. Photosynthesis linearly decreases with a decrease in soil water content for a rice variety that does not have any drought tolerance. With an increase in drought tolerance, photosynthesis would not have a significant reduction under minor drought stress (Fig. S3).

Figure S3. Illustration of the relationship of water stress to photosynthesis under different drought tolerance abilities.

#### *Nitrogen uptake*

Nitrogen uptake is coupled with water uptake (Fig. S4) and is determined by the plant water uptake rate and nitrogen concentration in the soil solution. Increasing soil water content dilutes nitrogen concentration and reduces the amount of nitrogen uptake with the same amount of water uptake.


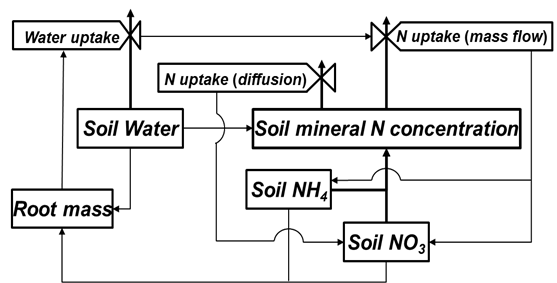


**Figure S4.** Illustration of the coupling scheme of water and nitrogen uptake. Solid boxes represent state variables while the tied valves represent rate variables. The solid arrows indicate mass flow while the thin lines represent the information input for computing state and rate variables.

This improved module in ORYZA2000 accounts for two types of nitrogen uptake: mass flow (or active uptake, *U_Nm_*, kg N ha^-1^ d^-1^) and diffusion (or passive uptake, *U_Nd_*, kg N ha^-1^ d^-1^). Total potential nitrogen uptake (*U_Np_*, kg N ha^-1^ d^-1^) is the sum of these two types of uptakes (Eqs. S73 and S74), which is quantified as maximum soil nitrogen supply under soil water and nitrogen conditions,

$U_{Np}=U_{Nm}+U_{Nd}$ (S73)

The term *U_Nm_* is the result of the water uptake rate and mineral nitrogen solution concentration,

$U_{Nm}=\sum_{i=1}^{n} u_{w,i}\frac{N_{s,i}}{10\theta_{i}z_{i}}$ (S74)

where *θ_i_* is the soil water content (cm^3^ cm^-3^) in the *i^th^* soil layer, *z_i_*, is the thickness of the layer (cm), *N_s,i_* is the mineral nitrogen content (kg N ha^-1^), *u_w,i_* is the water uptake rate (mm d^-1^), and *n* is the number of rooting soil layers.

With the same amount of mineral nitrogen in the soil, a higher water content will dilute the concentration and result in lower uptake per unit of water uptake (Fig. S5). However, *u_w,i_* will increase before it reaches the maximum uptake (potential transpiration), which can compensate for the reduction in dilution. *U_Np_* positively relates to the substance (mineral N) amount and accessibility (or diffusion pathway), which is defined as the fraction of the physically available water to the total amount of soil water (Eq. 29),

$U_{Nd}=\left\{ \begin{aligned} \sum_{i=1}^{n} \frac{\theta_{i}-\theta_{i}^{wp}}{\theta_{i}}\frac{N_{s,i}}{10\theta_{i}Z_{i}} (\theta_{i}>\theta_{i}^{wp}) \\ 0 (\theta_{i}\leq\theta_{i}^{wp}) \end{aligned} \right.$ (S75)

where $\theta_{i}^{wp}$ is the soil water content at the wilting point of the plant (cm^3^/cm). In equation S75, the term ($\frac{N_{s,i}}{10\theta_{i}z_{i}}$) is the same as the concentration of solution, but $\frac{1}{10\theta_{i}Z_{i}}$ is used as a unit-less dilution factor, which also implies a dilution effect for less nitrogen uptake because of a higher concentration gradient between the soil solution and root solution when soil water content is higher. The term $\frac{\theta_{i}-\theta_{i}^{wp}}{\theta_{i}}$ presents tortuosity effects on diffusion when soil water content is higher. Low tortuosity at the condition with higher soil water content results in higher diffusion uptake (Fig. S5).

**Figure S5.** The nitrogen mass flow and diffusion uptake of water and nitrogen content in the soil.

Actual nitrogen uptake is limited by soil supply (*U_Np_*, kg N ha^-1^ d^-1^), plant demand (*D_N_*, kg N ha^-1^ d^-1^), and maximum uptake capability (*U_Nmax_*, kg N ha^-1^ d^-1^) (Eq. S75). Demand depends on the amount of nitrogen needed to make up the maximum nitrogen concentration ($r_{j}^{'}$, kg N kg^-1^ dry mass) from the current concentration (*r_j_*, kg N kg^-1^ dry mass) in plant tissues (*j*, root, stem, leaf, and panicle),

$U_{N}=max(U_{Nmax},U_{Np},D_{N})$ (S76)

$D_{N}=max(0.0, \sum_{j=1}^{n} (r_{j}^{'}-r_{j})w_{j})$ (S77)

where parameter $r_{j}^{'}$ is a genetic parameter for nitrogen concentration in plant organs under an optimal growth environment.

#### *Nitrogen deficiency stress*

Nitrogen deficiency stress (*S_N_*) is quantified as the ratio of actual nitrogen uptake to the nitrogen demand. Nitrogen stress can slow down transpiration, resulting in a reduction in potential photosynthesis, but the stronger one between nitrogen deficiency stress and drought stress will be a factor in the reduction in transpiration and photosynthesis and will be used to adjust assimilate allocation among plant organs.

### Assimilate allocation among plant organs under drought and/or nitrogen deficiency stress

The assimilate allocation algorithm has been modified to take into account the effects of drought stress, nitrogen deficiency, and light competition on assimilate allocation. A group of modified functions, from the functions introduced by Friedlingstein et al. ([1999)](#_1fob9te) and used by Li et al. ([2009)](#_49x2ik5), is used to perform assimilate allocation in two steps: (1) splitting assimilates into two parts, for the root and for the shoot, respectively (Eqs. S78 and S79), and (2) splitting the assimilates for the shoot into two parts, for the leaf and stem during the vegetative stage, and three portions for the leaf, stem, and panicle during the reproductive stage (Eqs. S80 to S82):

$F_{sh}=\frac{F_{sh}^{'}}{1.0+f_{c}(1.0-f_{wn})}$ (S78)

$F_{r}=1.0-F_{sh}$ (S79)

$F_{st}=\frac{F_{st}^{'}+\frac{F_{st}^{'}}{F_{st}^{'}+F_{so}^{'}}f_{c}(1.0-f_{r)}+f_{c}(1.0-f_{wn})}{1.0+f_{c}(2.0-f_{r}-f_{wn})}$ (S80)

$F_{l}=\frac{F_{l}^{'}}{1.0+f_{c}(2.0-f_{r}-f_{wn})}$ (S81)

$F_{so}=\frac{F_{so}^{'}+\frac{F_{so}^{'}}{F_{st}^{'}+F_{so}^{'}}f_{c}(1.0-f_{r})}{1.0+f_{c}(2.0-f_{r}-f_{wn})}$ (S82)

In equations S78 to S82, the terms $F_{sh}^{'}$, $F_{st}^{'}$, $F_{so}^{'}$, and $F_{l}^{'}$ are the partitioning coefficients of a given variety under non-stress and optimal growth conditions, which are defined as genetic coefficients of the variety. The *f_c_* is a climatic correcting factor, which decreases by 0.1 per 15 degree increase in latitude from the equator (Eq. S83). As *f_c_* decreases with an increase in latitude (*L*, degree), more leaf area is needed to capture the same amount of radiation because of the lower sun angle at higher latitude:

$f_{c}=min(1.0, max(0.0, 1.0-\frac{0.1|L|-1.5}{15.0})$) (S83)

In equations S78 to S82, *f_r_* is the light competition factor which is determined by the light extinction coefficient (*γ*), the leaf area index (*LAI*), and the maximum leaf area index (*LAI_max_*) (Eq. S84). *LAI_max_* is the leaf area index above the light compensation point (*ρ*, 6.9 W/s for C_3_ rice) under instant radiation conditions (*R_d_*, J d^-1^) (Eq. S85):

$f_{r}=1.0-{(1.0-min (1.0,e^{\gamma({LAI}_{max}-LAI)}) )}^{\sigma}$ (S84)

${LAI}_{max}=max(0.0, -\frac{{ln}_{10}(\frac{7200\rho d_{l}}{R_{d}})}{\gamma})$ (S85)

where σ is the sensitivity of a rice variety to low light intensity which varies
from 2 to 6 and has a default value of 4, and *d_l_* is the day length of the effective photosynthetic period (hours) ([Ampong-Nyarko et al., 1992;](#_3rdcrjn) [Resurreccion et al., 2002; Venkateswarlu et al., 1977)](#_ihv636).

In equations S78 to S82, *f_wn_* is quantified as the minimum value of the water and nitrogen deficiency stress factor (Eq. S86). The value of 1.5 in this equation slows down the response to water or nitrogen stress because assimilate allocation is much less sensitive to the stress than photosynthesis:

$f_{wn}=(1.0-{(1.0-min (S_{w},S_{N}) )}^{1.5DTF})$ (S86)

With this modified algorithm of assimilate allocation, water and/or nitrogen stress promotes more assimilate to root rather than shoot, and more to leaf and storage rather than to stem in aboveground organs (Fig. S6). The plant could either establish more roots to explore a larger soil volume for water and/or nitrogen, have a larger leaf area to maintain productivity, or reduce consumption in the stem to ensure the size of storage.

Light competition results in more assimilate to stem and storage such that the plant could occupy a larger spatial volume to capture more radiation or reduce the size of its inefficient forage. The plant also pretends to maintain the size of storage under the light competition.

**Figure S6**. Assimilate allocation relating to water and/or nitrogen stress and light competition.

## Simulation results of case studies

The graphic and statistical analyses were employed to quantify the predictability of ORYZA (v3) in comparison with ORYZA2000 (v2.13). The data used in these analyses were time-series measurements and simulation outputs on the dates of measurements, and also the measured and simulated seasonal yields. The simulation outputs were either from ORYZA (v3) or ORYZA2000 (v2.13). The variables included these analyses were plant organ biomass, leaf area index, leaf nitrogen contents, and water potential at different soil layers (Table S1, Figs. S7 to S10).

**Table S1.** The statistical results for simulation outputs of ORYZA (v3) against the measurements in the calibration and validation datasets, respectively. The analysis as described in the section of statistical analysis are the regression, student T-test, error and modeling efficiency between simulated and measured values.

| Analyzed variable^[1]^ | **Pairs** | **Y** | **X** | **α** | **β** | **r^2^** | **P(t)** | **RMSEn (%)** | **M_eff_** |
| --- | --- | --- | --- | --- | --- | --- | --- | --- | --- |
|  | **Calibration dataset** | | | | | | | | |
| WAGT | 168 | 4.79 | 4.73 | 0.27 | 0.95 | 0.98 | 0.57 | 16.26 | 0.98 |
| WSO | 80 | 2.91 | 2.84 | 0.35 | 0.90 | 0.96 | 0.65 | 24.15 | 0.93 |
| WST | 168 | 1.88 | 1.87 | 0.29 | 0.85 | 0.91 | 0.14 | 25.82 | 0.92 |
| WLVD | 84 | 0.82 | 0.90 | 0.16 | 0.73 | 0.85 | 0.87 | 54.40 | 0.71 |
| WLVG | 168 | 1.06 | 1.03 | 0.16 | 0.88 | 0.92 | 0.70 | 35.79 | 0.87 |
| Yield | 30 | 4.37 | 4.55 | 0.27 | 0.90 | 0.95 | 0.81 | 16.33 | 0.90 |
| LAI | 168 | 2.17 | 2.04 | 0.68 | 0.73 | 0.89 | 0.95 | 0.37 | 0.83 |
| Leaf_N | 84 | 1.10 | 1.11 | 0.16 | 0.85 | 0.79 | 0.22 | 29.47 | 0.53 |
| AWD6 | 143 | 4.80 | 3.77 | -0.29 | 1.35 | 0.79 | 1.00 | 57.95 | -1.73 |
| AWD5 | 330 | 6.97 | 6.97 | -0.01 | 1.00 | 1.00 | 0.87 | 0.54 | 1.00 |
| AWD4 | 327 | 4.74 | 7.49 | 1.05 | 0.49 | 0.96 | 1.00 | 14.97 | 0.93 |
| AWD3 | 316 | 10.02 | 10.03 | -0.02 | 1.00 | 1.00 | 0.46 | 0.87 | 1.00 |
| AWD2 | 254 | 15.41 | 12.96 | 3.36 | 0.93 | 0.79 | 1.00 | 26.46 | 0.90 |
| ARE3 | 325 | 12.04 | 12.05 | 0.01 | 1.00 | 1.00 | 0.92 | 1.16 | 1.00 |
| AWD3+ARE3 | 641 | 11.05 | 11.05 | -0.01 | 1.00 | 1.00 | 0.90 | 0.79 | 1.00 |
|  | Validation dataset | | | | | | | | |
| WAGT | 173 | 4.72 | 4.76 | 0.28 | 0.93 | 0.98 | 0.41 | 18.73 | 0.97 |
| WSO | 81 | 2.87 | 2.91 | 0.25 | 0.90 | 0.96 | 0.39 | 23.98 | 0.93 |
| WST | 173 | 1.87 | 1.91 | 0.27 | 0.83 | 0.93 | 0.67 | 27.76 | 0.90 |
| WLVD | 91 | 0.79 | 0.83 | 0.15 | 0.78 | 0.85 | 0.52 | 53.67 | 0.72 |
| WLVG | 173 | 1.05 | 1.04 | 0.16 | 0.86 | 0.91 | 0.42 | 34.25 | 0.85 |
| Yield | 30 | 4.41 | 4.52 | 0.08 | 0.96 | 0.97 | 0.68 | 12.98 | 0.94 |
| LAI | 173 | 2.21 | 2.06 | 0.66 | 0.76 | 0.87 | 0.98 | 38.45 | 0.76 |
| LEAF_N | 84 | 1.08 | 1.09 | 0.25 | 0.76 | 0.77 | 0.41 | 30.06 | 0.53 |
| AWD6 | 213 | 5.93 | 5.00 | 0.93 | 1.00 | 0.72 | 1.00 | 55.97 | -0.34 |
| AWD5 | 309 | 6.77 | 6.78 | 0.01 | 1.00 | 1.00 | 0.68 | 0.68 | 1.00 |
| AWD4 | 306 | 4.73 | 7.79 | 1.51 | 0.41 | 0.95 | 1.00 | 28.63 | 0.70 |
| AWD3 | 288 | 10.24 | 10.27 | -0.01 | 1.00 | 1.00 | 0.99 | 1.35 | 1.00 |
| AWD2 | 226 | 17.08 | 17.03 | 4.80 | 0.72 | 0.65 | 0.04 | 66.47 | 0.50 |
| ARE3 | 329 | 12.55 | 12.55 | 0.00 | 1.00 | 1.00 | 0.74 | 0.59 | 1.00 |
| AWD3+ARE3 | 617 | 11.47 | 11.49 | -0.01 | 1.00 | 1.00 | 0.99 | 1.20 | 1.00 |

^[1]^: The crop growth variables involved in the statistical analysis were WAGT: total above-ground biomass (t ha^-1^), WSO: panicle biomass (t ha^-1^), WST: stem biomass (t ha^-1^), WLVG: green leaf biomass (t ha^-1^), LAI: leaf area index, Leaf_N: nitrogen content of green leaves (g N m^-2^ leaf), and Yield: grain yield (t ha^-1^). The soil variables used for statistical analysis were AWD2, AWD4, AWD5 and AWD6: soil water potential in the 2^nd^, 4^th^, 5^th^ and 6^th^ soil layers in AWD experiment, respectively, and AWD3 + ARE3: the water potential of the 3^rd^ soil layer in AWD and ARE experiments.

**
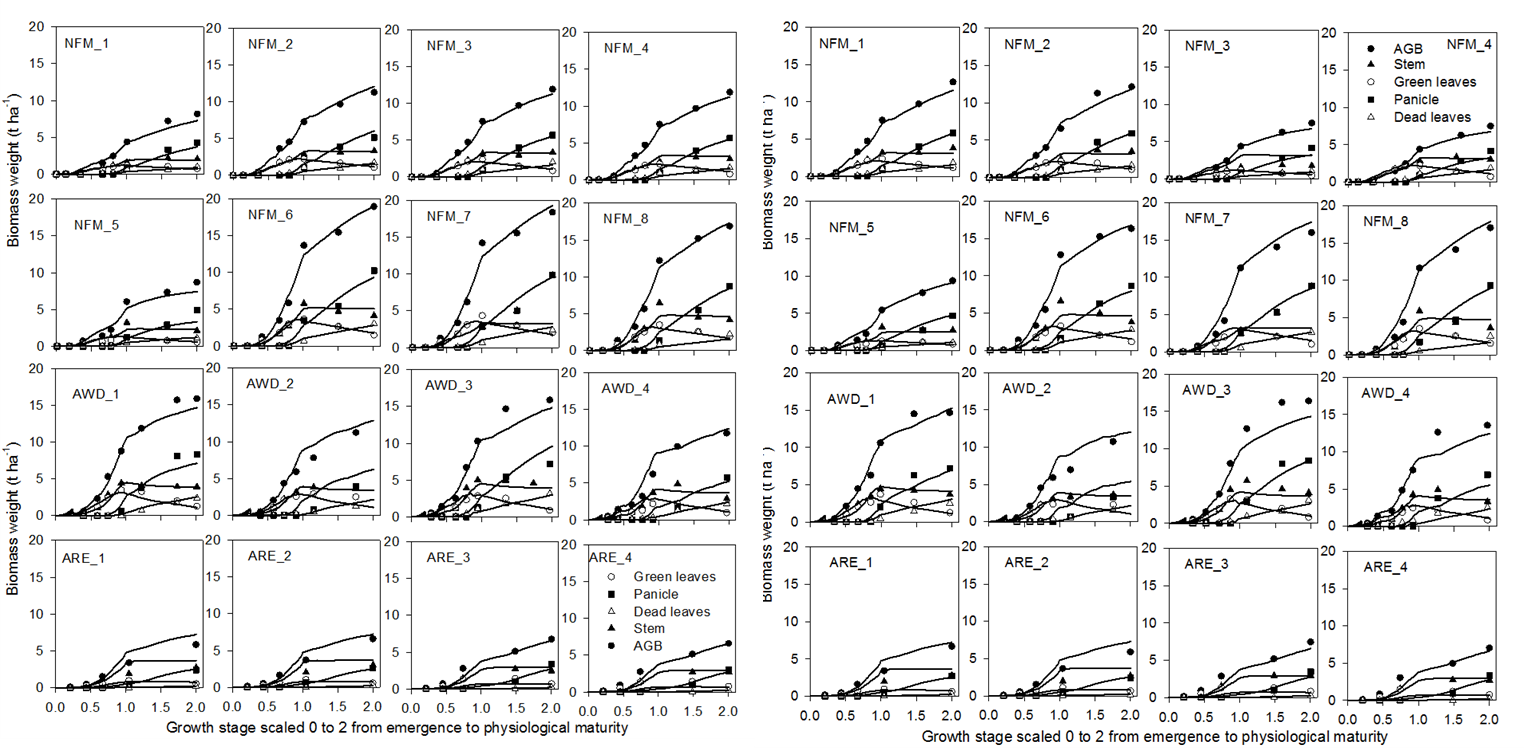
**

**Figure S7:** The simulated and measured organ biomass in a growth season using the calibration (left panel group) and validation (right panel group) datasets. The symbols are the measured values at different growth stages while the lines represent the simulated dynamic of organ biomass during the growth season. NFM_1 to NFM_8 are the treatments 1 to 8 of the nitrogen fertilizer management experiment (NFM) (Table 3). AWD_1 is the continuously flooded treatment, while AWD_2 to AWD_4 are the treatments W1 to W3 in water-limited experiment (AWD) (Table 2). ARE_1 to ARE_4 are the treatments W1N1, W2N1, W1N2 and W2N2 in the aerobic rice experiment (ARE) (Table 4).


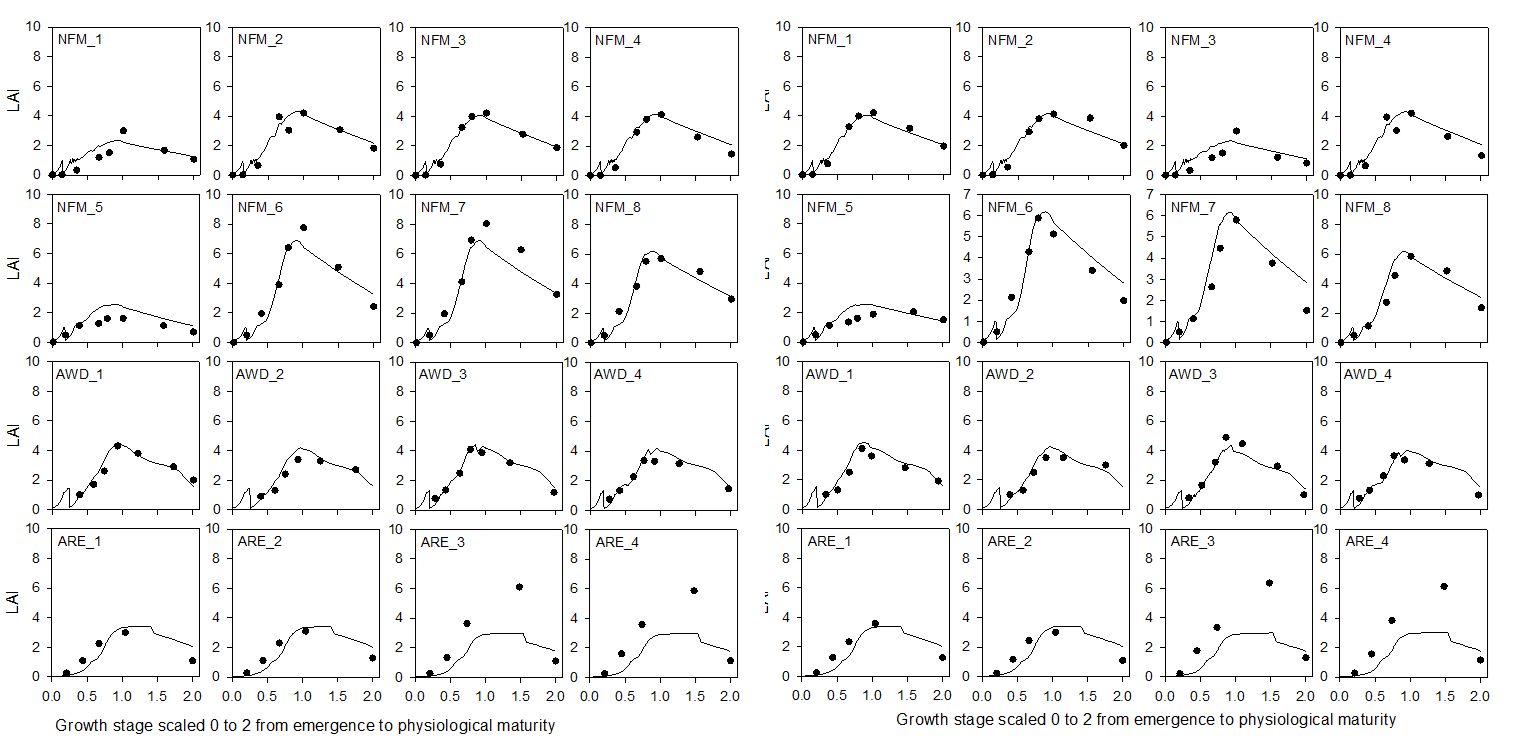


**Figure S8:** The simulated and measured leaf area index (LAI) in a growth season using the calibration (left panel group) and validation (right panel group) datasets. The symbols are the measured values at different growth stages while the lines represent the simulated dynamic of organ biomass in the growth season. NFM_1 to NFM_8 are the treatments 1 to 8 of the nitrogen fertilizer management experiment (NFM) (Table 3). AWD_1 is the continuously flooded treatment, while AWD_2 to AWD_4 are the treatments W1 to W3 in water-limited experiment (AWD) (Table 2). ARE_1 to ARE_4 are the treatments W1N1, W2N1, W1N2 and W2N2 in the aerobic rice experiment (ARE) (Table 4).


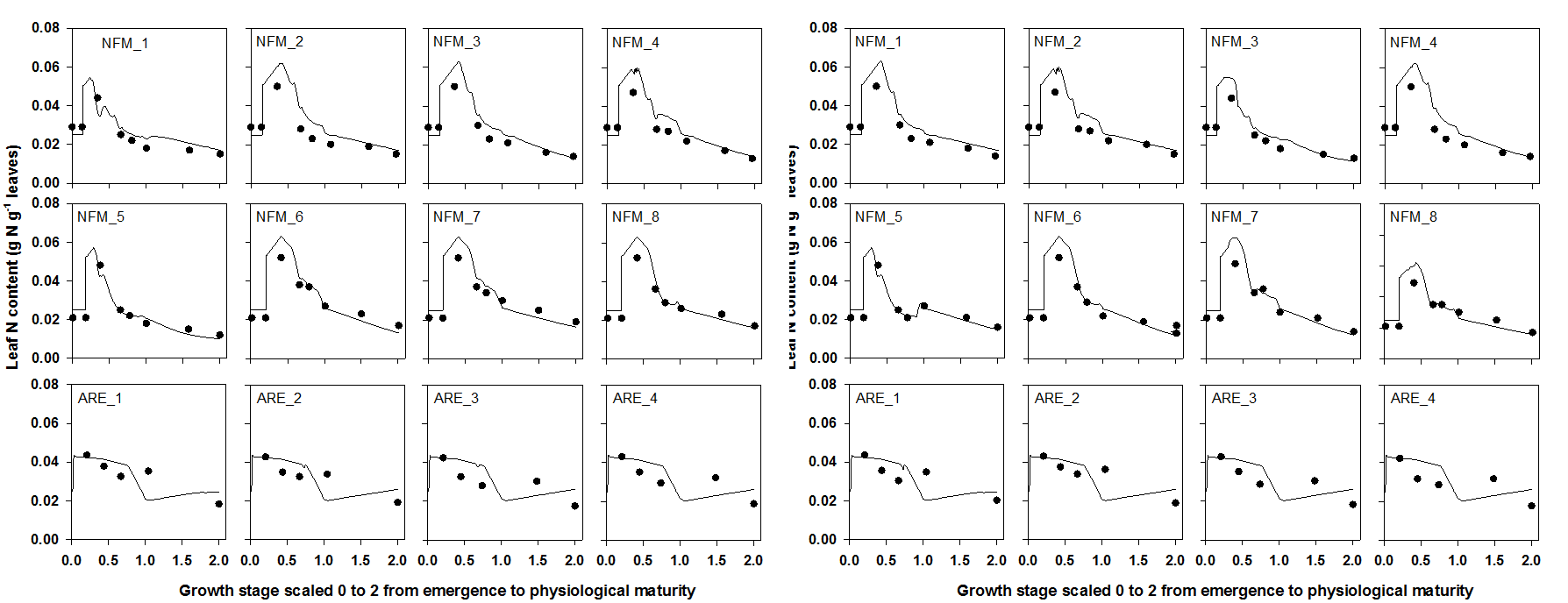


**Figure S9:** The simulated and measured leaf nitrogen contents in a growth season using the calibration (left panel group) and validation (right panel group) datasets. The symbols are the measured values at different growth stages while the lines represent the simulated dynamic of organ biomass in the growth season. NFM_1 to NFM_8 are the treatments 1 to 8 of the nitrogen fertilizer management experiment (NFM) (Table 3). ARE_1 to ARE_4 are the treatments W1N1, W2N1, W1N2 and W2N2 in the aerobic rice experiment (ARE) (Table 4).


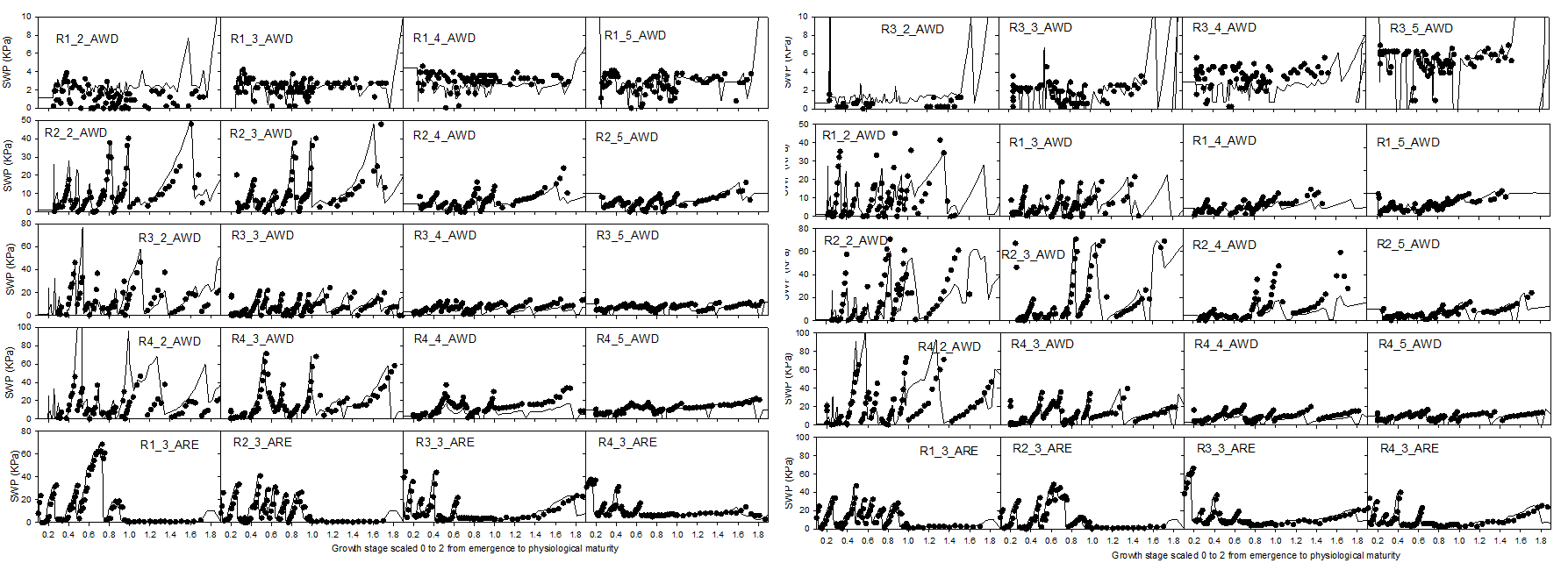


**Figure S10:** The simulated and measured soil water potential (SWP) in different soil layers in a growth season using the calibration (upper panel group) and validation (low panel group) datasets. The symbols are the measured values at different growth stages while the lines represent the simulated dynamic of organ biomass in the growth season. For the syntax of the label in each small panel, R1 to R4 indicate the water management schemes CF, W1, W2, and W3 in the water-limited experiment (AWD) (Table 2) and treatments W1N1, W2N1, W1N2 and W2N2 in the aerobic rice experiment (ARE) (Table 4), while the numbers (2 to 5) indicate the second to fifth soil layers below the soil surface.

## Simulation Inputs

**Table S2:** The values of crop parameters for the different cultivars in simulations of case studies. TC: Thermal controlled leaf growth ((^o^Cd)^-1^), SLA: special leaf area (ha kg^-1^), DRLV: leaf death coefficient (d^-1^), DTF: drought tolerance factor, KDF: light extinction coefficient, EFF: effect of air temperature on light use efficiency. FLV, FST, FSO and FSH are the proportion factors of assimilate allocated to leaf, stem, panicle and above ground shoot respectively.

| Parameter | | FFZ | GSR IR1-5-SAL10-DT1-DT1 | GSR IR1-8-SAL12-Y2-DT1 | GSR IR1-12-DT10-SAL1-DT1 | IR74371-70-1-1 | PSBRC82 | IR83142-B-19-B | IR72 | PAU201 | HD297 |
| --- | --- | --- | --- | --- | --- | --- | --- | --- | --- | --- | --- |
| Phenology | | 0.00058,0.00075,0.00204 | 0.00059,0.00075,0.00204 | 0.00063,0.00078,0.00177 | 0.00056,0.00075,0.00212 | 0.00058,0.00075,0.00179 | 0.00058,0.00065,0.00326 | 0.00069,0.00082,0.00189 | 0.00068,0.00075,0.00196 | 0.00058,0.00061,0.00207 | 0.00086,0.00101,0.00403 |
| Leaf development | TC | 0.0156,0.0064 | 0.0094,0.0038 | 0.0063,0.0027 | 0.0114,0.0036 | 0.0054,0.0043 | 0.0099,0.0031 | 0.0063,0.0036 | 0.0115,0.0048 | 0.0093,0.0042 | 0.0073,0.0038 |
|  | SLA | 0.0064,0.5835,0.0065,0.0026,0.0020,0.0036,0.0018 | 0.0038,0.5755,0.0067,0.0043,0.0027,0.0035,0.0017 | 0.0027,0.5835,0.0030,0.0054,0.0022,0.0026,0.0017 | 0.0036,0.5755,0.0043,0.0066,0.0036,0.0019,0.0020 | 0.0043,0.5835,0.0058,0.0048,0.0018,0.0031,0.0030 | 0.0031,0.4613,0.0065,0.0059,0.0019,0.0031,0.0029 | 0.0036,0.5546,0.0046,0.0029,0.0018,0.0023,0.0028 | 0.0048,0.6000,0.0016,0.0014,0.0014,0.0014,0.0014 | 0.0042,0.3530,0.0024,0.0014,0.0013,0.0014,0.0016 | 0.0038,0.1709,0.0045,0.0034,0.0053,0.0046,0.0046 |
|  | DRLV | 0.02,0.04,0.11,0.05,0.00,0.00 | 0.02,0.03,0.07,0.04,0.00,0.00 | 0.01,0.01,0.04,0.09,0.00,0.00 | 0.01,0.03,0.05,0.05,0.00,0.00 | 0.02,0.02,0.05,0.06,0.00,0.00 | 0.01,0.02,0.07,0.05,0.00,0.00 | 0.01,0.03,0.07,0.04,0.00,0.00 | 0.01,0.02,0.02,0.01,0.00,0.00 | 0.02,0.03,0.02,0.07,0.00,0.00 | 0.00,0.00,0.02,0.02,0.00,0.00 |
| Assimilation | DTF | 1.71 | 1.74 | 1.71 | 1.74 | 1.71 | 2.17 | 1.80 | 1.67 | 2.83 | 5.85 |
|  | KDF | 0.56,0.48,0.48,0.63 | 0.48,0.37,0.54,0.41 | 0.45,0.47,0.41,0.49 | 0.59,0.59,0.42,0.68 | 0.57,0.41,0.47,0.51 | 0.32,0.48,0.69,0.54 | 0.45,0.36,0.57,0.61 | 0.20,0.38,0.43,0.41 | 0.33,0.31,0.47,0.50 | 0.53,0.68,0.67,0.36 |
|  | EFF | 0.35,0.01,0.10,0.71,0.26,0.46 | 0.04,0.01,0.01,0.57,0.39,0.39 | 0.01,0.01,0.00,0.59,0.38,0.50 | 0.01,0.05,0.01,0.27,0.31,0.55 | 0.29,0.00,0.00,0.81,0.56,0.63 | 0.02,0.00,0.02,0.26,0.47,0.60 | 0.04,0.01,0.02,0.79,0.44,0.32 | 0.03,0.02,0.01,0.45,0.85,0.54 | 0.06,0.01,0.00,0.65,0.93,0.54 | 0.05,0.08,0.00,0.44,0.58,0.54 |
| Assimilate allocation | FST | 0.64,0.37,0.83,0.15,0.17,0.03 | 0.63,0.44,0.57,0.25,0.28,0.03 | 0.53,0.58,0.48,0.73,0.44,0.16 | 0.83,0.30,0.50,0.62,0.20,0.25 | 0.58,0.35,0.83,0.33,0.22,0.15 | 0.38,0.57,0.57,0.50,0.00,0.00 | 0.31,0.32,0.84,0.64,0.06,0.15 | 0.48,0.55,0.34,0.43,0.07,0.00 | 0.29,0.36,0.51,0.11,0.01,0.00 | 0.72,0.78,0.60,0.80,0.05,0.00 |
|  | FSO | 0.50,0.82,0.88 | 0.71,0.71,0.97 | 0.26,0.56,0.84 | 0.37,0.76,0.73 | 0.38,0.78,0.85 | 0.47,1.00,0.98 | 0.33,0.93,0.83 | 0.54,0.91,0.99 | 0.84,0.99,1.00 | 0.15,0.86,1.00 |
|  | FLV | 0.36,0.63,0.17,0.35,0.01,0.10 | 0.37,0.56,0.43,0.04,0.01,0.01 | 0.47,0.42,0.52,0.01,0.01,0.00 | 0.17,0.70,0.50,0.01,0.05,0.01 | 0.42,0.65,0.17,0.29,0.00,0.00 | 0.62,0.43,0.43,0.02,0.00,0.02 | 0.69,0.68,0.16,0.04,0.01,0.02 | 0.52,0.45,0.58,0.03,0.02,0.01 | 0.71,0.64,0.49,0.06,0.01,0.00 | 0.28,0.22,0.28,0.05,0.08,0.00 |
|  | FSH | 0.71,0.26 | 0.57,0.39 | 0.59,0.38 | 0.27,0.31 | 0.81,0.56 | 0.26,0.47 | 0.79,0.44 | 0.45,0.85 | 0.65,0.93 | 0.44,0.58 |

**Table S3.** The values of soil parameters used in the simulations of case studies. The NFM, AWD, ARE MVD are the codes of nitrogen fertilizer management, water management, aerobic rice and multiple variety drought experiments (Table 1).

| Experiment | NFM | AWD | ARE | MVD |
| --- | --- | --- | --- | --- |
| Soil layers | 6 | 9 | 7 | 7 |
| Thickness of layer (m) | 0.10,0.10,0.20,0.20,0.20,0.20 | 0.05,0.10,0.10,0.10,0.20,0.10,0.30,0.30,0.30 | 0.05,0.05,0.05,0.2,0.33,0.14,0.18 | 0.10,0.10,0.10,0.15,0.15,0.20,0.20 |
| Sand content | 0.53,0.53,0.49,0.46,0.45,0.45 | 0.44,0.47,0.47,0.47,0.46,0.46,0.22,0.18,0.12 | 0.53,0.53,0.53,0.47,0.36,0.36,0.39 | 0.35,0.35,0.31,0.33,0.30,0.31,0.30 |
| Clay content | 0.20,0.20,0.26,0.30,0.31,0.30 | 0.36,0.38,0.38,0.38,0.40,0.40,0.40,0.42,0.46 | 0.07,0.07,0.07,0.18,0.35,0.37,0.35 | 0.35,0.35,0.31,0.33,0.30,0.31,0.30 |
| Saturated hydraulic conductivity  (cm d^-1^) | 11.32,11.32,5.87,3.70,3.28,3.64 | 150.00,150.00,100.00,100.00,100.00,100.00,100.00,100.00,100.00 | 474.12,8.56,210.28,10.85,8.56,517.16,10.85 | 89.86,89.74,107.33,1.05,3.98,41.84,43.54 |
| Soil organic carbon content (t ha^-1^) | 11.58,11.58,14.15,9.47,8.37,6.65 | 9.62,7.30,3.14,2.68,4.35,1.73,5.21,5.58,5.01 | 6.62,6.62,6.62,2.27,2.71,0.77,0.91,5.58,5.01 | 11.06,10.93,8.46,9.32,7.52,8.72,6.99 |
| Saturated water content (cm^3^cm^-3^) | 0.39,0.39,0.40,0.41,0.41,0.41 | 0.47,0.47,0.46,0.47,0.47,0.47,0.48,0.49,0.49 | 0.38,0.38,0.38,0.39,0.43,0.43,0.43 | 0.42,0.42,0.41,0.36,0.41,0.41,0.41 |
| Water content at field capacity (cm^3^cm^-3^) | 0.23,0.23,0.27,0.29,0.30,0.30 | 0.38,0.38,0.37,0.39,0.39,0.39,0.40,0.41,0.41 | 0.16,0.16,0.16,0.22,0.33,0.35,0.33 | 0.32,0.33,0.30,0.26,0.30,0.30,0.30 |
| Water content at wilting point  (cm^3^ cm^-3^) | 0.12,0.12,0.16,0.18,0.19,0.18 | 0.23,0.23,0.22,0.24,0.24,0.24,0.25,0.27,0.27 | 0.04,0.04,0.04,0.11,0.20,0.21,0.21 | 0.21,0.21,0.19,0.16,0.18,0.19,0.18 |

## Abbreviations in the supplementary

| **Abbreviation** | **Description** |
| --- | --- |
| *i* | order of soil layer |
| *t* | denotes time (seconds) |
| *T_i_* | soil temperature (^o^C) |
| *D_h,i_* | thermal diffusivity (m^2^ s^-1^) |
| *λ_i_* | thermal conductivity (J s^-1^ m^-1^ k^-1^) |
| *c_h.i_* | volumetric specific heat of the soil (J m^-3^ k^-1^) |
| *z_i_* | depth (m) of the i^th^ soil layer |
| *Δt* | time increment |
| *j* | superscript indicating the time at which temperature is determined |
| 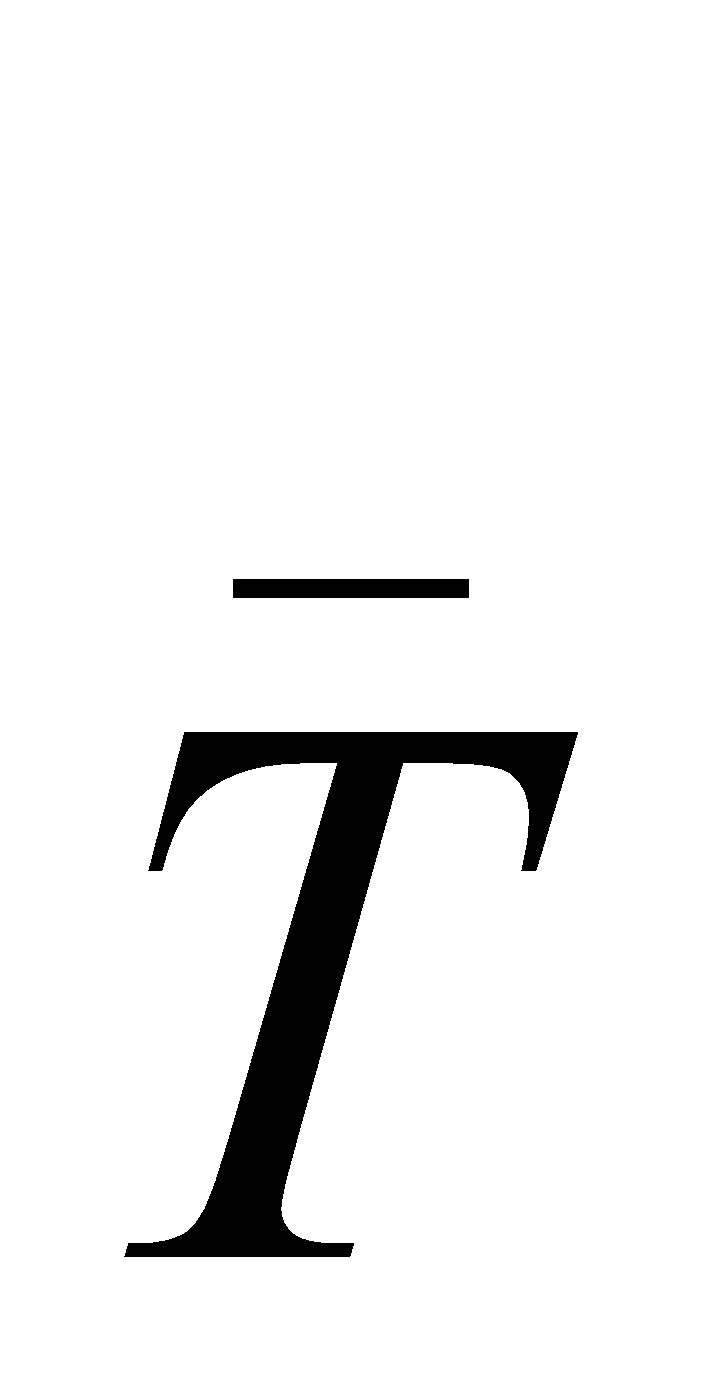 | appropriate mean temperature (^o^C) |
| *K_i_* | thermal conductance (J s^-1^ m^-2^ k^-1^) |
| *T^j^* | temperature at present time (^o^C) |
| *T^j+1^* | temperature at the next time step (^o^C) |
| *η* | weighing factor that ranges from 0 to 1.0 |
| *TN_i_* | new temperature (^o^C) |
| *D_i_* | boundary temperature (^o^C) |
| 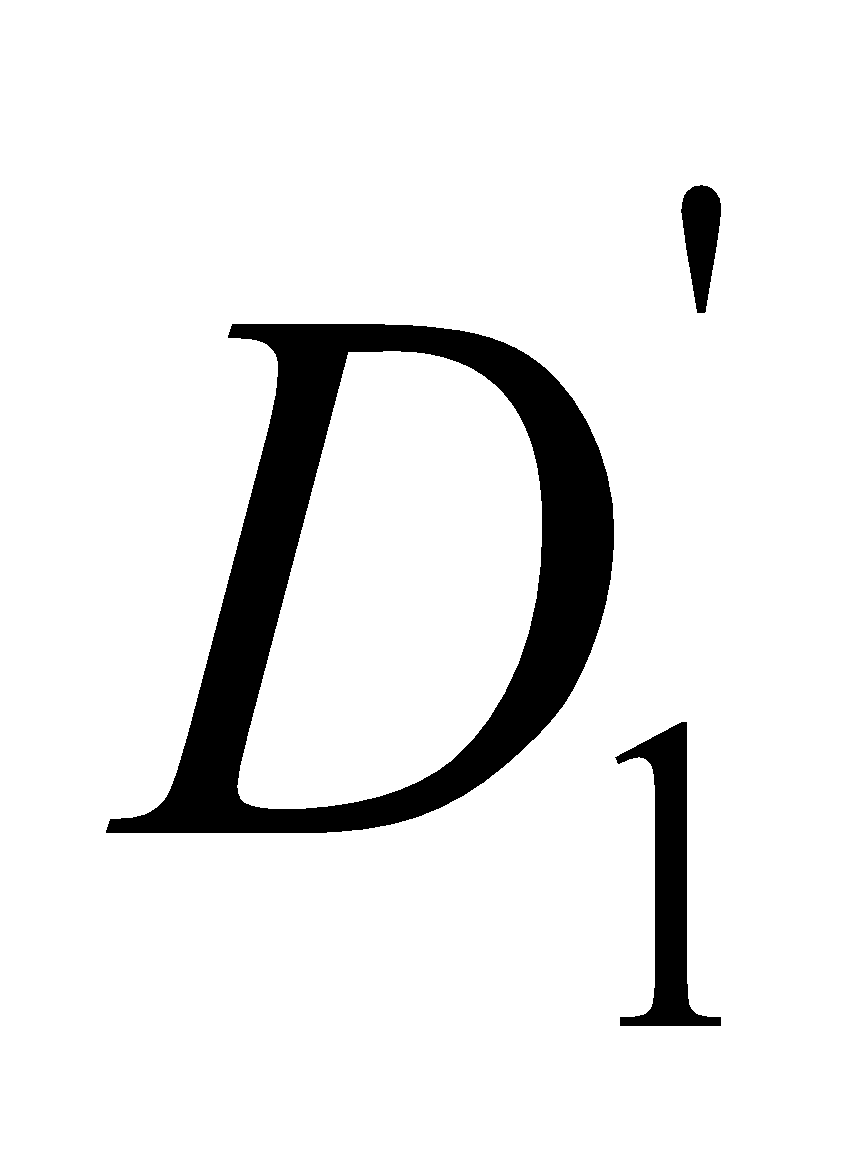 | upper boundary temperature at the previous time step (^o^C) |
| *T_a_* | present air temperature (^o^C) |
| *R_n_* | net radiation (J s^-1^ m^-2^) reaching the system surface |
| *LE* | latent heat flux (J s^-1^ m^-2^) |
| *T_i+1_* | set as the average annual temperature of soil (^o^C) |
| *B_i_* | soil bulk density (Mg m^-3^) |
| *C_i_^clay^* | fraction of clay content (g g^-1^) |
| *C_t_* | fresh organic carbon content (g C m^-3^) at time t |
| *C_t−1_* | fresh organic carbon content in an earlier time step (g C m^-3^) |
| *K_t_* | actual decomposition rate (d^-1^) |
| *δ_t_* | time step |
| *k* | potential decomposition rate (d^-1^) |
| *f(T_s_)* | function of soil temperature (*Ts*, ^o^C) |
| *f(θ)* | function of soil water content (*θ*, cm^3^ cm^-3^) |
| *f(R_CN_)* | function of the organic matter C to N ratio (*R_CN_*) used to modify carbon decomposition and mineralization |
| *θ_FC_* | soil water content at field capacity (cm^3^ cm^-3^) |
| *θ_r_* | residual soil water content (cm^3^ cm^-3^) |
| *θ_s_* | saturated soil water content (cm^3^ cm^-3^) |
| *R_CN_* | C:N ratio of fresh organic matter |
| *R_CNmax_* | maximum C:N ratio of stabilized humus |
| *δ_c_* | total carbon decomposed (g C m^-3^); or new root carbon in a soil layer stratified from the total new root carbon on a given day (kg C ha^-1^) |
| 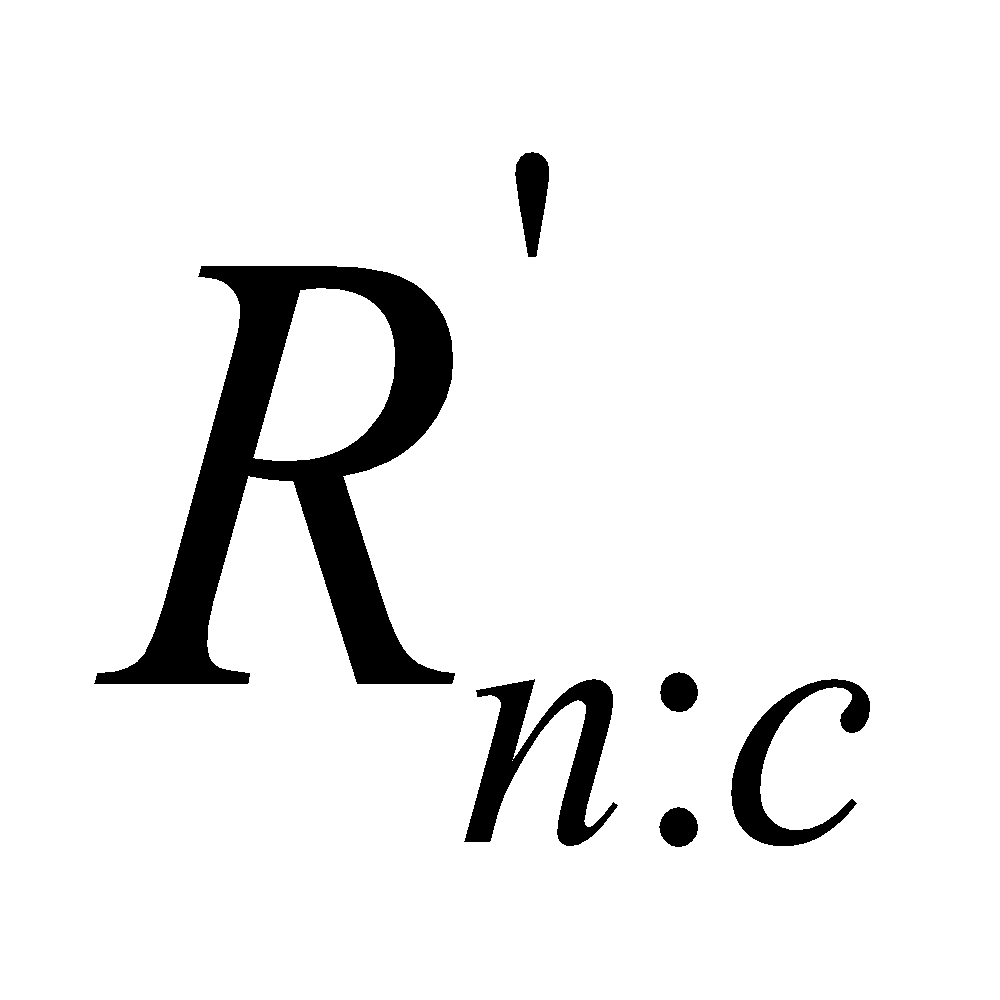 | N:C ratio of stable soil organic matter (1:11 normally) |
| *C_t−1_* | is the carbon content in fresh organic matter at a previous time (g C m^-3^) |
| *N_t – 1_* | nitrogen content in fresh organic matter at a previous time (kg N m^-3^) |
| *N_NH4_* | ammonium- nitrogen content in soil (g N m^-3^) |
| *N_NO3_* | nitrate-nitrogen content in soil (g N m^-3^) |
| *N^’^_NH4_* | reserve amounts of ammonium-nitrogen that the plant and microbes could not uptake (g N m^-3^) |
| *N^’^_NO3_* | reserve amounts of nitrate-nitrogen that the plant and microbes could not uptake (g N m^-3^) |
| *N_NH4,t_* | ammonium content in current time steps (kg N m^-3^) |
| *N_NO3,t_* | nitrate content in current time steps (kg N m^-3^) |
| *N_NH4,t-1_* | ammonium content in previous time steps (kg N m^-3^) |
| *N_NO3,t−1_* | nitrate content in previous time steps (kg N m^-3^) |
| *H_[Urea]_* | hydrolyzed amount (g N m^-2^) |
| *[Urea]* | content of urea (g N m^-2^) |
| *[NH_4_]* | content of ammonium (g N m^-2^) |
| *f_[Urea]_* | potential hydrolyzing rate (g N m^-2^) |
| *f_w_* | modified function of soil water to hydrolysis |
| *f_T_* | modified function of soil temperature to hydrolysis in a soil layer; factor of soil temperature to scale down the nitrification rate in the soil layer |
| *pH* | pH of a soil layer |
| *TOC* | Total soil organic carbon (g C m^-2^) |
| *h^T^_1_* | compensation temperature coefficient for hydrolysis at low temperature |
| *h^T^_2_* | maximum soil temperature for active hydrolysis (^o^C) |
| *z* | thickness (m) of a soil layer |
| *d* | depth (m) of a soil layer |
| *f_d_* | factor of soil depth to scale down the nitrification rate |
| *T^s^* | soil temperature (^o^C) to scale down the nitrification rate in a soil layer |
| *C_w_* | corresponding coefficient between soluble carbon and denitrification in a soil layer (g C m^-2^) |
| *R_N2/N2O_* | ratio of N_2_ to N_2_O in nitrogen gas |
| *f_r_(NO_3_)* | function to determine the ratio of N_2_ to N_2_O by nitrate content (kg N m^-2^) |
| *f_r_(CO_2_)* | function to determine the ratio of decomposed carbon (g C m^-2^) |
| *f_r_(θ)* | function to determine the ratio of soil water content in a soil layer |
| *R_c_* | total carbon allocated into the root from assimilate (kg C ha^-1^) |
| *A_c_* | daily accumulated root carbon allocated (kg C ha^-1^) |
| D_c_ | total senescence (kg C ha^-1^) in the soil layer |
| 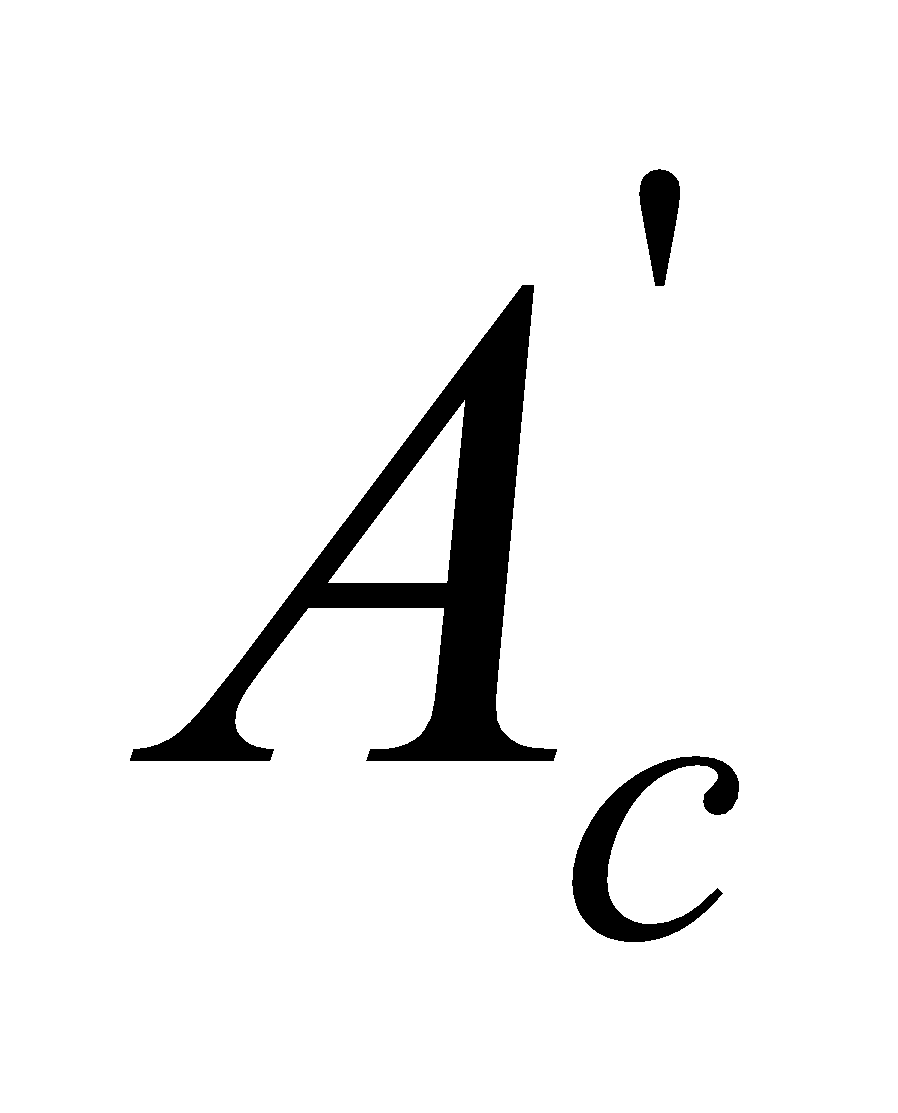 | allocated root carbon on the previous day (kg C ha^-1^) |
| *f_i_* | distribution factor (unit-less) in the soil layer *i* |
| *δ_c,i_* | specified *δ_c_* (new root carbon) for the soil layer *i* (kg C ha^-1^) |
| *n* | number of rooted soil layers |
| *d_x,i_* | depth of calculation from the soil surface (cm) |
| *x* | changes from the upper to the lower boundary of soil layer *i* |
| *k* | extinction coefficient of the new root distribution in the soil profile |
| *p_i_* | soil penetration factor that influence root distribution |
| *t_i_* | temperature factor that influence root distribution |
| *w_i_* | water factor that influence root distribution |
| *n_i_* | nitrogen factor that influence root distribution |
| *DVS* | development stage ranging from 0 to 2 |
| *d^’^* | transplanting or direct-seeding depth (cm) |
| *d_max_* | genetic parameter representing the maximum rooting depth of a variety (cm) |
| *d_max_ DVS* | the moment potential depth of the rooting frontier in the vegetative stage, which increases parallel to plant development (cm) |
| *t_i_* | temperature suitability factor ranging from 0 to 1.0 and the layer with the most suitable temperature for root growth is 1.0 |
| 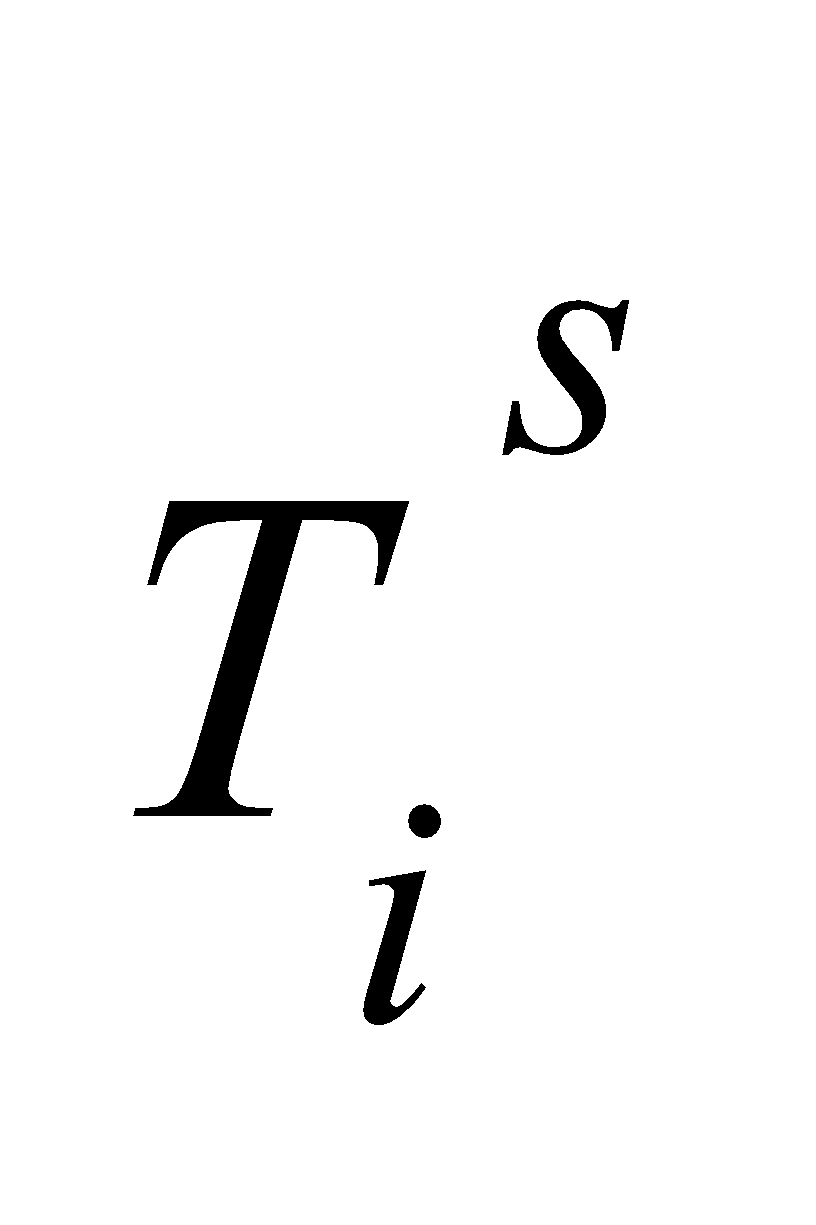 | soil temperature in the i^th^ soil layer (^o^C) |
| *T_i_* | index of the most suitable soil temperature for root growth |
| 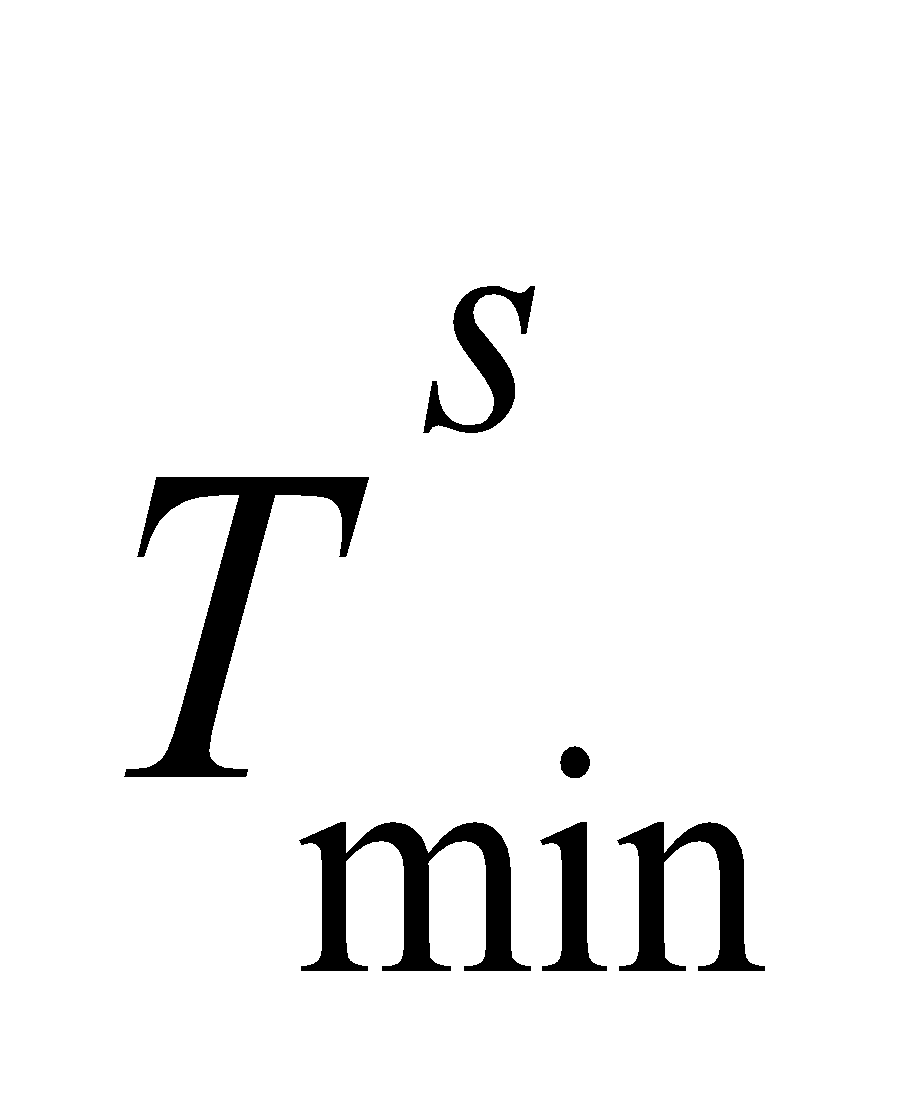 | lowest soil temperature for root survival (^o^C) |
| 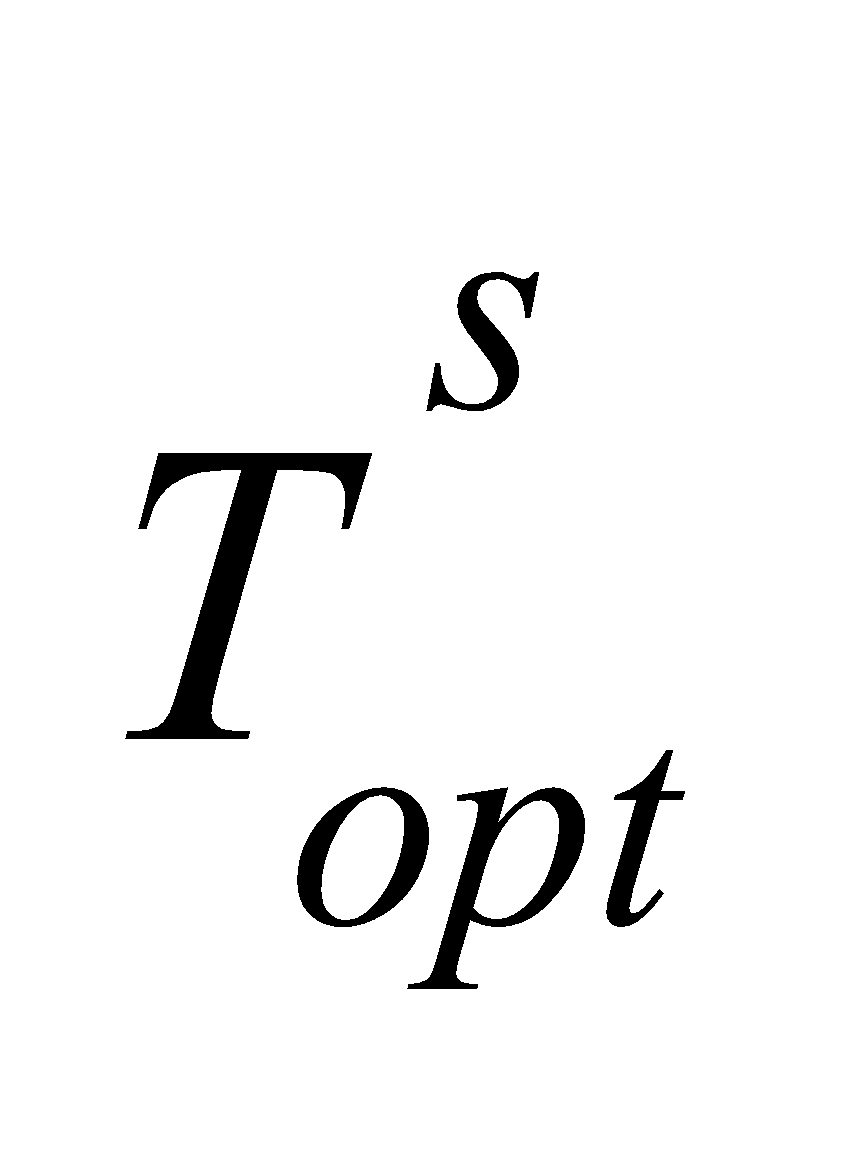 | optimum soil temperature (^o^C) |
| 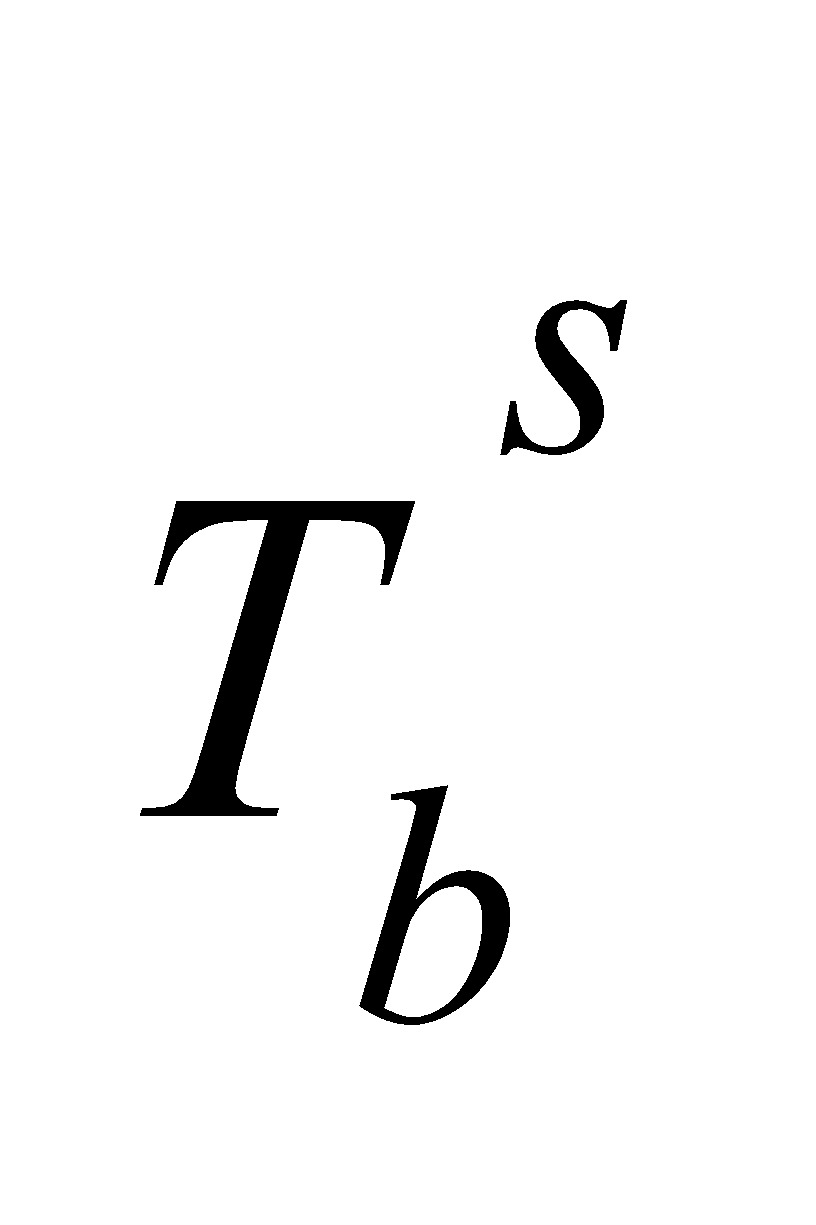 | lowest soil temperature (^o^C) |
| *T_max_* | maximum soil temperature (^o^C) |
| *a_i_* | air factor for rice root growth in i^th^ soil layer |
| *A_i_* | air effect on the root growth in soil layer *i* |
| *A_max_* | maximum value of all *A_i_* within the rooted soil layers |
| *F^’^* | ability to tolerate saturated conditions |
| *S_i_^w^* | ratio of the volumetric soil water content to the total porosity of soil layer *i* |
| *F_i_^w^* | critical water-filled porosity of soil layer *i* |
| *θ_i_* | volumetric soil water content (cm^3^ cm^-3^) |
| *θ_i_^s^* | total porosity of soil layer *i* |
| *p_i_* | factor of soil penetrability |
| *SP_i_* | penetrability of the root in soil layer *i* (MPa^-1^) |
| *SP_max_* | maximum of all *SP_i_* within rooted soil layers (MPa^-1^) |
| *P_i_^’^* | initial soil penetration resistance (MPa) |
| *w_i_* | moisture effect on root growth in soil layer *i* |
| *W_max_* | maximum *W_i_* among all values within root soil layers |
| *θ_j_^wp^* | wilting point of soil layer *i* (cm^3^ cm^-3^) |
| *N_i_* | mineral nitrogen content in soil layer *i* (kg N ha^-1^) |
| *N_max_* | maximum *N_i_* within the rooted soil layers (kg N ha^-1^) |
| SWCST | saturated soil water content |
| SWCWP | Wilting point soil water content |
| DTF | threshold at which transpiration will decline significantly if soil water content decreases continuously towards SWCWP |
| *θ_x_* | variable in the integration function for soil water content varying between the integrating range |
| *U_w,p_* | potential uptake of the whole rooting profile (mm) |
| *R_c,i_* | root biomass of soil layer *i* (kg C/ha) |
| *U_w_* | actual uptake of water (mm) |
| *T_p_* | potential transpiration (mm) |
| *S_w_* | drought stress index |
| *U_Nm_* | mass flow or active uptake (kg N ha^-1^ d^-1^) |
| *U_Nd_* | diffusion or passive uptake (kg N ha^-1^ d^-1^) |
| *U_Np_* | total potential nitrogen uptake (kg N ha^-1^ d^-1^) |
| *N_s,i_* | mineral nitrogen content (kg N ha^-1^) |
| *D_N_* | plant nitrogen demand (kg N ha^-1^ d^-1^) |
| *U_Nmax_* | maximum uptake capability (kg N ha^-1^ d^-1^) |
| $r_{j}^{'}$ | genetic parameter for nitrogen concentration in plant organs under an optimal growth environment (kg N kg^-1^ dry mass) |
| *S_N_* | nitrogen deficiency stress |
| $F_{sh}^{'}$ | partitioning coefficient (shoot) of a given variety under non-stress and optimal growth conditions |
| $F_{st}^{'}$ | partitioning coefficient (stem) of a given variety under non-stress and optimal growth conditions |
| $F_{so}^{'}$ | partitioning coefficient (storage organ) of a given variety under non-stress and optimal growth conditions |
| $F_{l}^{'}$ | partitioning coefficient (leaf) of a given variety under non-stress and optimal growth conditions |
| *f_c_* | climatic correcting factor |
| *f_r_* | light competition factor which is determined by the light extinction coefficient |
| *γ* | light extinction coefficient |
| *LAI* | leaf area index (m^2^ m^-2^ land) |
| *LAI_max_* | maximum leaf area index (m^2^ m^-2^ land) |
| *ρ* | light compensation point (W s^-1^) |
| *R_d_* | instant radiation conditions (j d^-1^) |
| σ | sensitivity of a rice variety to low light intensity |
| *d_l_* | day length of the effective photosynthetic period (hours) |
| *f_wn_* | minimum value between the water and nitrogen deficiency stress factor |
| SWP | soil water potential (kpa) |
| AWD | alternate wetting and drying |
| CF | continuously flooded |

## References

Ampong-Nyarko, K., De Datta, S.K., Dingkuhn, M., 1992. Physiological response of rice and weeds to low light intensity at different growth stages. Weed Research 32, 465-472.

Azhar, A.H., Perera, B.J.C., 2006. Modelling water uptake by plants using improved algorithms. Pakistan Journal of Water Resources 10, 9.

Benjamin, J.G., Nielsen, D.C., Vigil, M.F., 2003. Quantifying effects of soil conditions on plant growth and crop production. Geoderma 116, 137-148.

Busscher, W.J., Bauer, P.J., 2003. Soil strength, cotton root growth and lint yield in a southeastern USA coastal loamy sand. Soil and Tillage Research 74, 151-159.

Campbell, S.G., 1985. Soil physics with basic: transport models for soil – plant systems. Elsevier Science Publishing Compant Inc. New York. Pp26- 39.

Canarache, A., 1990. PENETR — a generalized semi-empirical model estimating soil resistance to penetration. Soil and Tillage Research 16, 51-70.

Chaudhary, T.N., Chopra, U.K., Sinha, A.K., 1985. Root growth, leaf water potential and yield of irrigated summer mung bean (Phaseolus aureus roxb.) In relation to soil water status and soil temperature under various mulches. Field Crops Research 11, 325-333.

Del Grosso, S.j., Parton, W.J., Mosier, A.R., Ojima, D.S., Kulmala, A.E., Phongpan S., 2000. General model for N2O and N2 gas emissions from soils due to dentrification. Global Biogeochemical Cycles. 14, 1045-1060.

Drew, M.C., 1983. Plant injury and adaptation to oxygen deficiency in the root environment: A review. Plant and Soil 75, 179-199.

Feng, Y., Li, X., 2001. An Analytical Model of Soil Organic Carbon Dynamics Based on A Simple "Hockey Stick" Function. Soil Science 166, 431-440.

Friedlingstein, P., Joel, G., Field, C.B., Fung, I.Y., 1999. Toward an allocation scheme for global terrestrial carbon models. Global Change Biology 5, 755-770.

Gerard, C.J., Sexton, P., Shaw, G., 1982. Physical Factors Influencing Soil Strength and Root Growth1. Agronomy Journal. 74, 875-879.

Godwin, D.C., Jones, C.A., 1991. Nitrogen dynamics in soil-plant systems. American Society of Agronomy, Inc., Madison, pp. 287-321.

Grable, A.R., 1966. Soil Aeration and Plant Growth. In: Norman, A.G. (Ed.), Advances in Agronomy. Academic Press, pp. 57-106.

Jakobsen, B.F., Dexter, A.R., 1987. Effect of soil structure on wheat root growth, water uptake and grain yield. A computer simulation model. Soil and Tillage Research 10, 331-345.

Jones, C.A., Bland, W.L., Ritchie, J.T., Williams, J.R., 1991. Simulation of root growth. American Society of Agronomy, Inc., Madison, pp. 91-123.

Kirschbaum, M.U.F., 1995. The temperature dependence of soil organic matter decomposition, and the effect of global warming on soil organic C storage. Soil Biology and Biochemistry 27, 753-760.

Laboski, C.A.M., Dowdy, R.H., Allmaras, R.R., Lamb, J.A., 1998. Soil strength and water content influences on corn root distribution in a sandy soil. Plant and Soil 203, 239-247.

Lampurlanés, J., Angás, P., Cantero-Martı́nez, C., 2001. Root growth, soil water content and yield of barley under different tillage systems on two soils in semiarid conditions. Field Crops Research 69, 27-40.

Li, T., Feng, Y., Li, X., 2009. Predicting crop growth under different cropping and fertilizing management practices. Agricultural and Forest Meteorology 149, 985-998.

Lipiec, J., Arvidsson, J., Murer, E., 2003. Review of modelling crop growth, movement of water and chemicals in relation to topsoil and subsoil compaction. Soil and Tillage Research 73, 15-29.

Lipiec, J., Hatano, R., 2003. Quantification of compaction effects on soil physical properties and crop growth. Geoderma 116, 107-136.

McInnes, K., 1981. Thermal Conductivities of Soils from Dryland Wheat Regions of Eastern Washington. Washington State University.

Micucci, F.G., Taboada, M.A., 2006. Soil physical properties and soybean (Glycine max, Merrill) root abundance in conventionally- and zero-tilled soils in the humid Pampas of Argentina. Soil and Tillage Research 86, 152-162.

Resurreccion, A.P., Makino, A., Bennett, J., Mae, T., 2002. Effect of light intensity on the growth and photosynthesis of rice under different sulfur concentrations. Soil Science and Plant Nutrition 48, 71-77.

Rolston, D.E., Sharoley, A.N., Toy, D.W., Hoffman, D.L., Broadbent, F.E., 1980. Denitrification as affected by irrigation frequency of a field soil. EPA -600/2-80-06 U.S. Environmental Protection Agency, ADA, Oklahoma, USA.

Ronda, R.J., de Bruin, H.A.R., Holtslag, A.A.M., 2001. Representation of the Canopy Conductance in Modeling the Surface Energy Budget for Low Vegetation. Journal of Applied Meteorology 40, 1431-1444.

Salih, A.A., Babikir, H.M., Ali, S.A.M., 1998. Preliminary observations on effects of tillage systems on soil physical properties, cotton root growth and yield in Gezira Scheme, Sudan. Soil and Tillage Research 46, 187-191.

Schenk, H.J., 2008. The Shallowest Possible Water Extraction Profile: A Null Model for Global Root Distributions. Vadose Zone Journal 7, 1119-1124.

Simonson, J.R., 1975. Engineering heat transfer. Macmillan Press. London.

Stone, J.A., Taylor, H.M., 1983. Temperature and the Development of the Taproot and Lateral Roots of Four Indeterminate Soybean Cultivars1. Agronomy Journal. 75, 613-618.

Venkateswarlu, B., Prasad, V.V.S.S., Rao, A.V., 1977. Effects of low light intensity on different growth phases in rice (Oryza sativa L.). Plant and Soil 47, 37-47.

Vepraskas, M.J., Miner, G.S., 1986. Effects of Subsoiling and Mechanical Impedance on Tobacco Root Growth1. Soil Science Society of America Journal. 50, 423-427.

Vigil, M.F., Kissel, D.E., 1995. Rate of nitrogen mineralized from incorporated crop residues as influenced by temperature. Soil Science Society of America Journal, 59, 1636-1644.

Vigil, M. E, Kissel, D. E., 1991. Equations for estimating the amount of nitrogen mineralized from crop residues. Soil Science Society of America Journal, 55, 757-761.

Vigil, M.F., Kissel, D.E., Smith, S.J., 1991. Field crop recovery and modeling of nitrogen mineralized from labeled sorghum resides. Soil Science Society of America Journal, 55:1031-1037.

Voorhees, W.B., Allmaras, R.R., Johnson, C.E., 1981. Alleviating temperature stress. In: Modifying the root environment to reduce crop ctress. Eds. Arkin G.E. and Taylor H.M. American Society of Agricultural Engineers, Michigan, USA. Pp. 217-266.

Watson, G.W., Kelsey, P., 2006. The impact of soil compaction on soil aeration and fine root density of Quercus palustris. Urban Forestry & Urban Greening 4, 69-74.

Wopereis, M.C.S., Kropff, M.J., Maligaya, A.R., Tuong, T.P., 1996. Drought-stress responses of two lowland rice cultivars to soil water status. Field Crops Research 46, 21-39.

Wu, W., Lu, H., Liu, W., Devare, M., Thies, J.E., Chen, Y., 2009. Decomposition of Bacillus thuringiensis ( Bt ) transgenic rice residues (straw and roots) in paddy fields. Journal of Soils and Sediments 9, 10.

Wulfsohn, D., Gu, Y., Wulfsohn, A., Mojlaj, E.G., 1996. Statistical analysis of wheat root growth patterns under conventional and no-till systems. Soil and Tillage Research 38, 1-16.

Zou, C., Penfold, C., Sands, R., Misra, R., Hudson, I., 2001. Effects of soil air-filled porosity, soil matric potential and soil strength on primary root growth of radiata pine seedlings. Plant and Soil 236, 105-115.
